# Yeast Midas’ touch: recent advances in the valorisation of methanol by yeasts

**DOI:** 10.1007/s11274-025-04642-x

**Published:** 2025-10-29

**Authors:** Alessandra Mauri, Lorenzo García Tejada, Lars M. Blank

**Affiliations:** 1 Institute of Applied Microbiology (iAMB), RWTH Aachen University, Aachen, Germany; 2 Corbion Innovation Center, Gorinchem, Netherlands; 3 WSS Research Centre “Catalaix”, Aachen, Germany

**Keywords:** Methanol, Methylotrophic yeast, Bioeconomy, C1 metabolism, Industrial bioprocessing, Land-free biotechnology

## Abstract

Traditionally, methanol has been used as a building block for a plethora of applications. It holds a key role in the chemical industry to produce paints, fabric, solvents, and plastics, and in producing biodiesel as the alcohol counterpart in the transesterification of plant oils and animal fats. Recently, methanol regained attention in industrial biotechnology due to its potential as an alternative substrate. Specifically, methanol is natively metabolised by a group of microorganisms called methylotrophs. Endowed with peculiar physiology, these can utilise the reduced C1 molecule as their sole carbon and energy source. While efforts to implement methylotrophic metabolism in heterologous hosts are still in its infancy, genetic engineering of methylotrophic yeast already succeeded in the conversion of methanol into high-value compounds, such as organic acids or recombinant proteins, boosting their potential as cell factories. Moreover, current technology developments allow for methanol synthesis from biomass or CO_2_ hydrogenation using green H_2_, thus envisioning from the latter a land-free biotechnology and an effective way of closing the carbon loop. This review presents the current state of the art for the biosynthesis of chemical building blocks from methanol by methylotrophic yeasts alongside an outlook on its potential within a sustainable economy.

**Graphical abstract:**

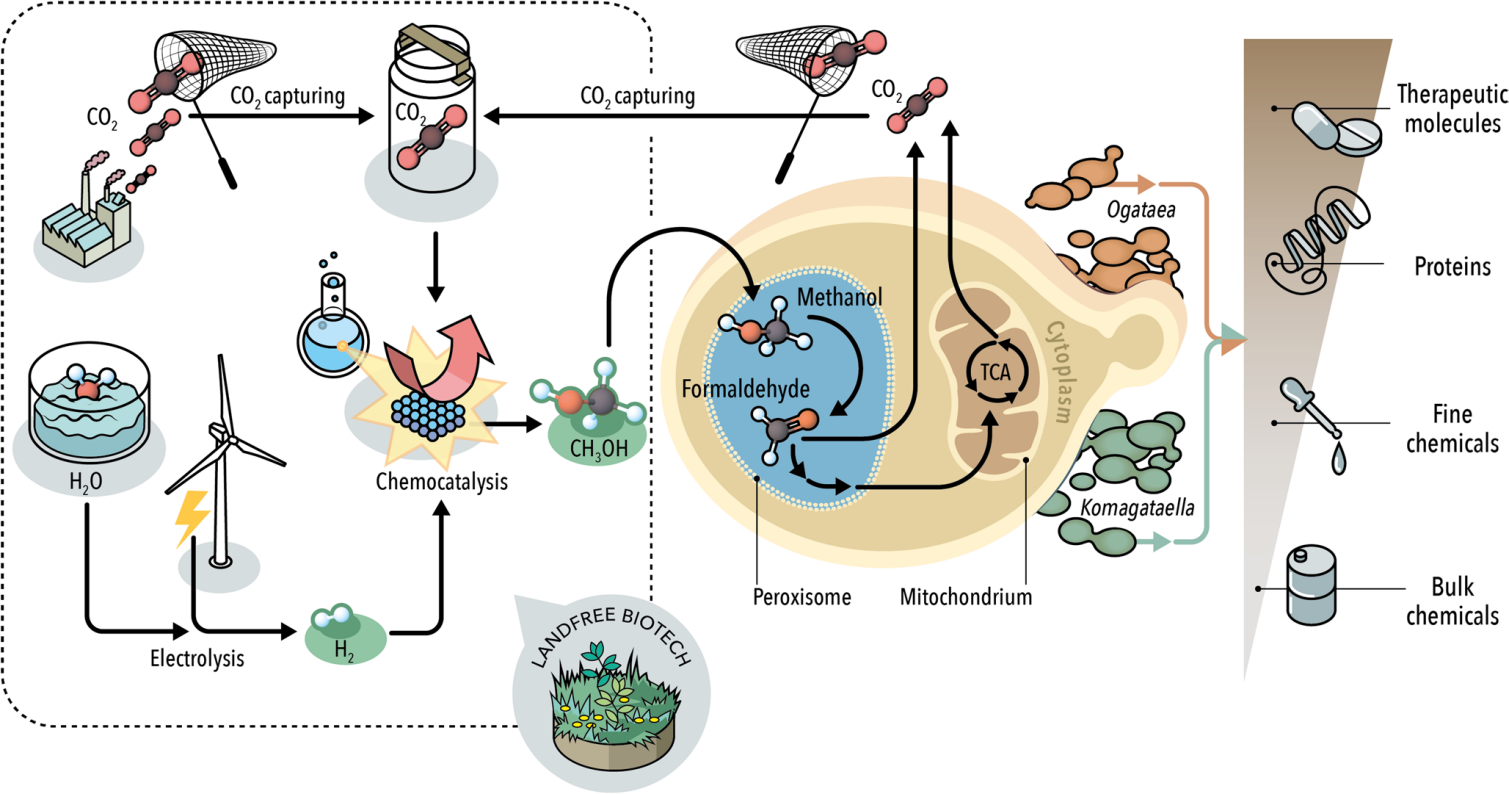

## Introduction: envisioning the revolution of a “land-free” biotechnology

Yeast fermentations using sugar as carbon and energy source are dominating food and biofuel production and contribute to the production of many other products. Land use for sugar production, which will be discussed later, is a central aspect for the future of microbial biotechnology. As an anecdote, the increase from 5% to 10% bioethanol in gasoline was a major communication disaster in Germany, with claims that the local bread roll will increase in price, with the consequence that 15 years later E10 fuels are still lower in sale despite an up to 5% price advantage. Many of us remember the tortilla crisis from 2007 in Mexico, although later debunked for the direct connection to bioethanol production in the USA (Keleman and Rañó 2012). With the absolute need to defossilise the chemical industry to meet CO_2_ reduction targets, the use of microbial biotechnology will massively increase, as we partly see already in China. There, with world leading companies in many of the microbially produced products, from vitamins to bulk amino acids, from specialty chemicals to bulk carboxylic acids, the amount of sugar fermented is in the double-digit percent of total sugar available, raising concern of further growth. While sugars dominate the recent growth in fermentation capacities, the research and to some extent production, is exploring other carbon and energy sources, such as CO_2_ + H_2_ (Bernal-Cabas et al. 2025; Daniell et al. 2012; Panich et al. 2021), plastic monomers (Liu et al. 2025; Tiso et al. 2021; Welsing et al. 2025), acetate (Ullmann et al. 2021; Ziegler et al. 2024), and as here discussed, methanol. In the short run, methanol will not contribute to the agreed-on Paris Climate goals, as it is synthesized from fossil resources such as gas or oil or in China even from coal, causing a significant CO_2_ footprint. However, with cheap methanol available that is competitive with sugar prices, firms can invest in the technology and can further optimise the value chain to reduce cost. In the mid to long term, green methanol, i.e., methanol produced from CO_2_ and green hydrogen will be available. The prerequisite is sustainable electricity from wind and solar or other routes like water dams, to split water into O_2_ and the required green H_2_. The point sources of the future for CO_2_ are expertly discussed elsewhere, while we would like to mention that the O_2_ from water splitting will intensify the fermentation industry, as higher production rates will be possible using oxygen enriched air. Safety measures must be taken into account, though. Green methanol as carbon and energy source for biotechnology can be seen as a carbon capture and utilization (CCU) technology. While companies like Solarfoods (https://solarfoods.com) showcase the technological feasibility of direct air capture for biotechnology, the economics argue clearly for CO_2_ point sources, such as biogas plants and cement kiln. Compared to other CCU uses, the replacement of sugar by green methanol comes with a stark reduction in land use, which comes close the “land free” biotechnology. Instead of huge farmland use for corn, sugar beet and sugar cane, land is mainly required for solar panels and windmills, while biogas and water dams contribute to energy storage possibilities. In Europe, farmland use for direct food production is below 25% and, world-wide, on average below 35%, while feed production is as high as 60%. A change in human diet from meat and dairy to more plant-based would allow increased land use for biotechnology, without challenging food security. However, with ever improving wind and solar technologies that are predicted to allow electricity production at rates as low as 1.5 dollar cents per kWh in favourable world regions, like south Chile or some parts of the Arabian peninsula, the here presented “land free” biotechnology using green methanol will become a reality. Ideally, some of the agricultural land is used for renaturing to slow down and finally stop the loss of biodiversity.

We here briefly introduce the current advances and advantages of green methanol as a carbon source for bioprocesses, followed by the metabolic pathways required for it, before summarizing literature examples, and ending with a weighted outlook on the intriguing carbon source methanol.

## Methanol, an established building block and commodity

Methanol (CH_3_OH), or methyl alcohol, is the simplest alcohol and a fundamental building block in the chemical industry. Interest in the molecule rose in the 1980 s to utilise it as a fuel alternative or as a platform chemical for chemical conversion onto other simple molecules, such as formaldehyde, acetic acid, methyl methacrylate, and methylamines (IRENA & Methanol Institute 2021; Olah 2005). Another application for methanol resides on its utilization as feedstock for bioprocesses. As a reduced C1 molecule with a lower oxidation state than glucose or sucrose and thus a higher energy density per molecule, it can be utilized as both energy and carbon source for microorganisms. This ability to utilize methanol has been long known for and studied in bacteria (Abou-Zeid and Baghlaf 1983; Söhngen 1905). Methanol metabolism in a yeast species was first described only in the late 1960 s, synchronously to the oil boom (Ogata et al. 1969). This research was founded on the economical availability of petrochemical compounds, with microbial protein production from reduced C1 compounds materializing in the form of single-cell protein (SCP) for food and feed supply (Abou-Zeid and Baghlaf 1983; Wegner 1990). However, the fluctuating prices of these feedstocks combined with cheaper product alternatives rendered methanol-based processes unfeasible for the production of SCP or bulk chemicals, and only the processes that produced high added value products like enzymes remained relevant (Cereghino et al. 2002; Macauley-Patrick et al. 2005).

Methanol is mainly produced using fossil sources in industrial settings, i.e., from synthesis gas, which is in turn derived from natural gas or coal (Olah 2005). Given the current desire to step away from non-renewable energy sources, the increasing availability of the solvent as experienced in the last decades of the twentieth century is highly compromised. Despite the syngas-based process being the gold standard, non-renewable feedstocks are responsible for GHG emissions and experience high volatility in their price and market due to their finite nature. Therefore, new feedstocks have been investigated, and innovative processes have been successfully developed to produce methanol in a greener, i.e., more sustainable way (Table 1). Based on the production process, different classes of methanol can be distinguished. Green methanol is generated by processes of renewable origin, like biomass gasification, or electrolysis fuelled by renewable electricity, whereas blue methanol is produced from non-renewable processes from natural gas with Carbon Capture Storage (CCS) (Madejski et al. 2022). Grey and brown methanol are exclusively produced through non-renewable processes utilizing natural gas and gasified coal, respectively, without CCS. The technologies employed to produce methanol can be summarized in two approaches: conversion through the catalysis of syngas, and the hydrogenation of carbon dioxide (CO_2_). In the first approach, either natural gas under steam reformation, gasified coal or biomass exposed to higher temperatures, are converted into syngas, a mixture of carbon monoxide and molecular hydrogen. This gas mixture is then further catalysed into methanol. The second approach involves a direct methanol synthesis (DMS) from CO_2_ and molecular hydrogen through the utilisation of a catalyst. In Table 1 we provide a comprehensive overview of the industrial processes currently known to produce methanol. Methanol is here classified based on the origin of the feedstocks and the synthesis process, along with the chemical reactions and the catalysts involved (Roy et al. 2018; Gautam et al. 2020; Shanmugam et al. 2021).

**Table 1:** Overview of the different feedstocks and feedstock origin, respective processes and specifications currently in use to produce methanol industrially

Feedstock	Origin	Process	Reactions	Catalysts	Type of CH_3_OH
Natural gas	Fossil	Steam methane reforming, methanol synthesis CCUS	$\:{CH}_{4}\:+\:{H}_{2}O\:\to\:\:CO\:+3{H}_{2}$ $\:CO+2{H}_{2}\to\:C{H}_{3}OH\:$ $\:{CO}_{2}\:+\:3{H}_{2}\:\to\:\:C{H}_{3}OH\:+{H}_{2}O$	Ni Cu/ZnO/Al₂O₃	grey, blue
Coal	Fossil	Gasification of coal, methanol synthesis	$\:C\:+\:O_2\:\to\:\:CO\:/\:CO_2$ $\:C\:+\:H_2O\:\to\:\:CO\:+\:H_2$ $\:{CO\:+\:2{H}_{2}\:\to\:\:CH}_{3}OH$ $\:{CO}_{2}\:+\:3{H}_{2}\:\to\:\:C{H}_{3}OH\:+{H}_{2}O$	Cu/ZnO/Al_2_O_3_ Cu/ZnO/Al_2_O_3_	brown
CO_2_ + green H_2_	Captured	Catalytic hydrogenation	$\:{CO}_{2}\:+\:3{H}_{2}\:\to\:\:C{H}_{3}OH\:+{H}_{2}O$	Cu/ZnO/Al₂O₃	green (e-)
CO_2_	Captured	Photocatalytic conversion	$\:C{O}_{2}\:+\:6{H}^{+}\:+\:6{e}^{-}\:\to\:C{H}_{3}OH\:+\:{H}_{2}O$	TiO_2_/Pt, Cu-TiO_2_	grey, green (e-)
CO_2_	Captured	Electrocatalytic reduction	$\:C{O}_{2}\:+\:6{H}^{+}\:+\:6{e}^{-}\:\to\:C{H}_{3}OH\:+\:{H}_{2}O$	Pt, Pd, Ru/Na- or K-modified β-alumina	grey, green (e-)
Biomass	Industrial waste	Gasification of biomass, methanol synthesis	$\:{C}_{x}{H}_{y}{O}_{z}\:+\:{O}_{2}/{H}_{2}O\:\to\:CO\:+\:H2+\:CO2\:+\:CH4$ $\:{CO\:+\:2{H}_{2}\:\to\:\:CH}_{3}OH$ $\:{CO}_{2}\:+\:3{H}_{2}\:\to\:\:C{H}_{3}OH\:+{H}_{2}O$	Fe–Cr, Cu–Zn Cu/ZnO/Al₂O₃	green (bio-)

## Old vs. new feedstocks: methanol and the “land-free” revolution

**Fig. 1:**
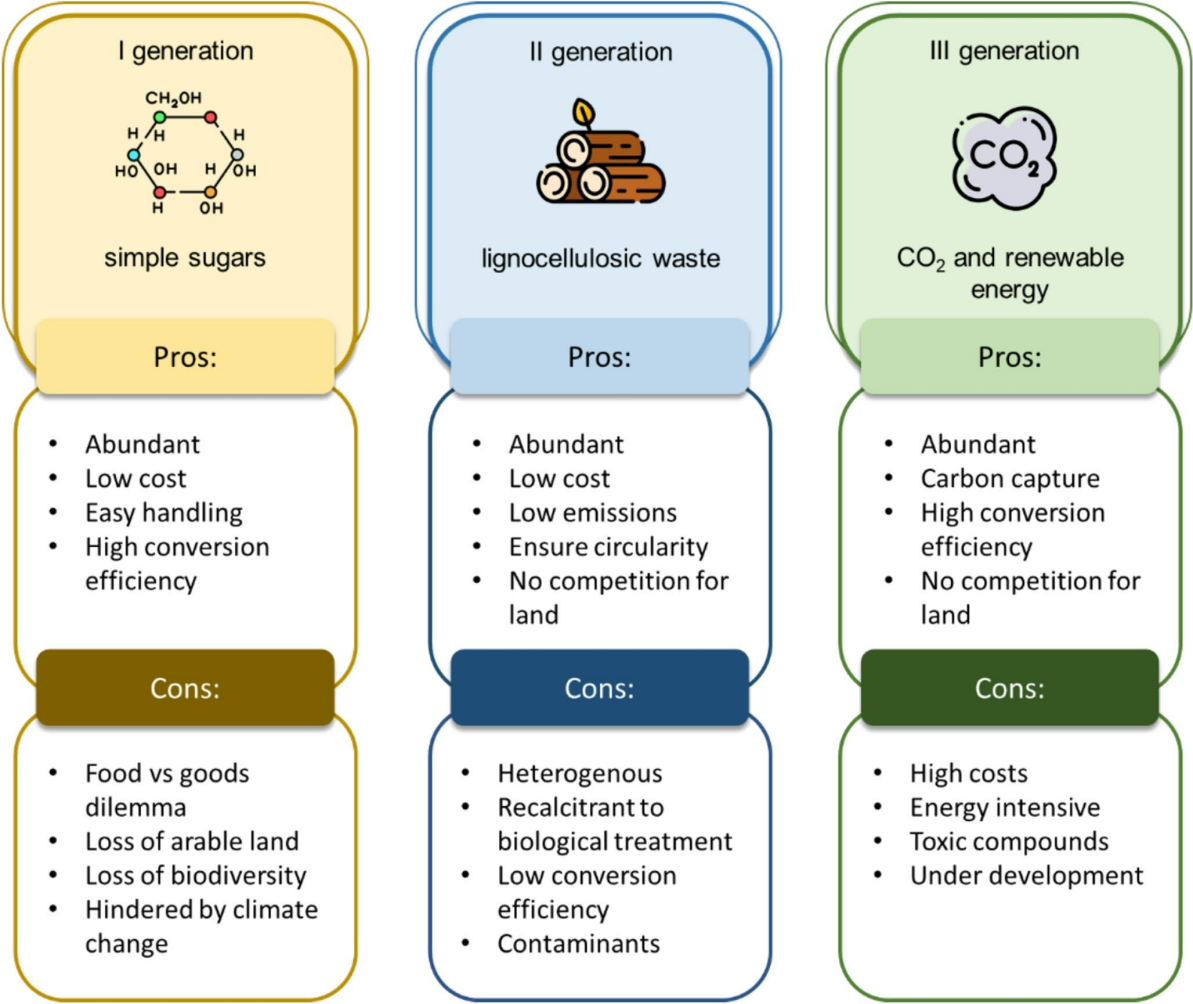
Schematic comparison of first, second and third generation feedstocks

Since the 1970 s, when its synthesis from methane became inexpensive, paving the way for yeast biomass and high-protein animal feed production, methanol has been regarded as an ideal fermentation substrate (Higgins and Cregg 1998). Due to the price increase of natural gas that plagued the same decade, the industry shifted its focus to other substrates, which were inexpensive and had high availability, such as sugar-based feedstocks. Nevertheless, using these feedstocks for non-food purposes, e.g., biofuels or chemical production, poses an ethical dilemma (Srinivasan 2009). Moreover, the biodiversity loss linked to extensive land usage is a further side effect (Oakley and Bicknell 2022). Hence, the industry’s interest partly shifted to second-generation (2G) feedstocks, which are mainly represented by lignocellulosic biomass. Despite not competing with the food sector, second-generation feedstocks present non-negligible drawbacks (Monlau et al. 2012). Due to its heterogeneous composition, lignocellulosic biomass processing is time- and energy-demanding. Logistics is highly challenging and requires new and highly developed local infrastructures (Monlau et al. 2012; Si et al. 2016). Also, the heterogeneous composition increases processing cost as non-fermentable residues must be removed either up- or downstream of the fermentation process, which is particularly challenging for non-volatile products (Fig. 1).

Third-generation (3G) feedstocks are hoped to reduce processing cost, preserve land, water and biodiversity, and tackle greenhouse gas emissions (GHG). These feedstocks are represented by captured carbon CO_2_ coupled with the use of renewable energies, such as electricity generated by wind power and photovoltaic cells (Liu et al. 2020, 2023). Atmospheric CO_2_ is an abundant feedstock: its levels have risen rapidly over the last century, reaching over 420 ppm in 2022, significantly exceeding the 350 ppm limit set at the Paris Climate Conference in 2015 (Garcia et al. 2022; NASA, Global Climate Change 2025). Hence, CCS from power-plant streams, industrial streams, or the atmosphere is a prominent approach and benefits from a well-established technology that addresses two major issues at the same time: alternative feedstock provision and GHG emissions. The fate of the carbon captured is double: it can be utilised (CCU) as a 3G feedstock to produce goods or it can be stored in geological sinks (CCS) until future utilisation. Comprehensively, this technology is known as Carbon Capture Utilisation and Storage (CCUS) (Garcia et al. 2022).

Despite significant technological advancement, the industrial production of green methanol is still not competitive compared to the fossil fuel-derived methanol and presents limitations. Specifically, the industrialisation bottlenecks are: (i) costly and complex carbon-free H_2_ supply, (ii) low process productivity and selectivity and (iii) catalysts performance limitations (Lais et al. 2018; Ye et al. 2019). Current costs of green hydrogen via electrolysis are typically $4–6 per kg, compared to $1–1.5 per kg for fossil-based hydrogen (Global Hydrogen Review 2022). Since hydrogen accounts for up to 70% of green methanol’s production cost, this cost disparity represents the most critical barrier to competitiveness. Furthermore, large-scale deployment would require massive renewable electricity inputs, as producing 1 ton of methanol consumes ~ 10–12 MWh (Kang et al. 2021). Conventional Cu-ZnO catalysts typically achieve methanol selectivity of 30–70% at CO_2_ conversion below 30% under standard reaction conditions. To address these limitations, new catalyst formulations, such as Cu–ZnO–Al_2_O_3_ and In-based materials, were developed showing improvement in selectivity, but unvaried methanol yields (Roy et al. 2018). Furthermore, at the plant-scale productivity is typically 0.1–0.5 g MeOH/g catalyst*h, with only a few catalysts reaching 1.0 g/g catalyst*h (Wesner et al. 2023; Araújo et al. 2024). Collectively, these three bottlenecks restrict the scalability and economic viability of green methanol production. Nonetheless, the production of green methanol is expected to pave the way to a “land-free” scenario, a term we aim to introduce in this review. Coupling goods production and use of alternative feedstock would mitigate land shortages for food and feed, while addressing GHG emissions. Moreover, a “land-free” approach would promote sustainable development and environmental stewardship in an era of growing awareness for resource conservation.

## Methanol metabolism and regulation

As an alcohol with low molecular weight, methanol poses toxicity issues to microbial cells, i.e., by modifying the fluidity of the cell membrane and interfering with the stability of proteins inside (Bennett et al. 2021; Wang et al. 2020). Inside the cell, it can be also enzymatically converted into compounds like formaldehyde or formic acid, which also pose toxicity to cell physiology. Formic acid inhibits the cytochrome c oxidase of the electron transport chain in certain yeast and bacterial species, causing a disbalance in the acid-base equilibrium and sending the cells into oxidative stress and, ultimately, apoptosis (Du et al. 2008; Kassem et al. 2017; Lee et al. 2010). Formaldehyde exerts a stronger toxicity in cells by performing protein and DNA cross-linking, which leads to impaired DNA replication, protein translation and reduced protein activity, sends cells into stress response and ultimately causes cell death (Chen et al. 2016; Jayakody and Jin 2021; Wani and Jain 2024; Yu et al. 2015).

**Fig. 2:**
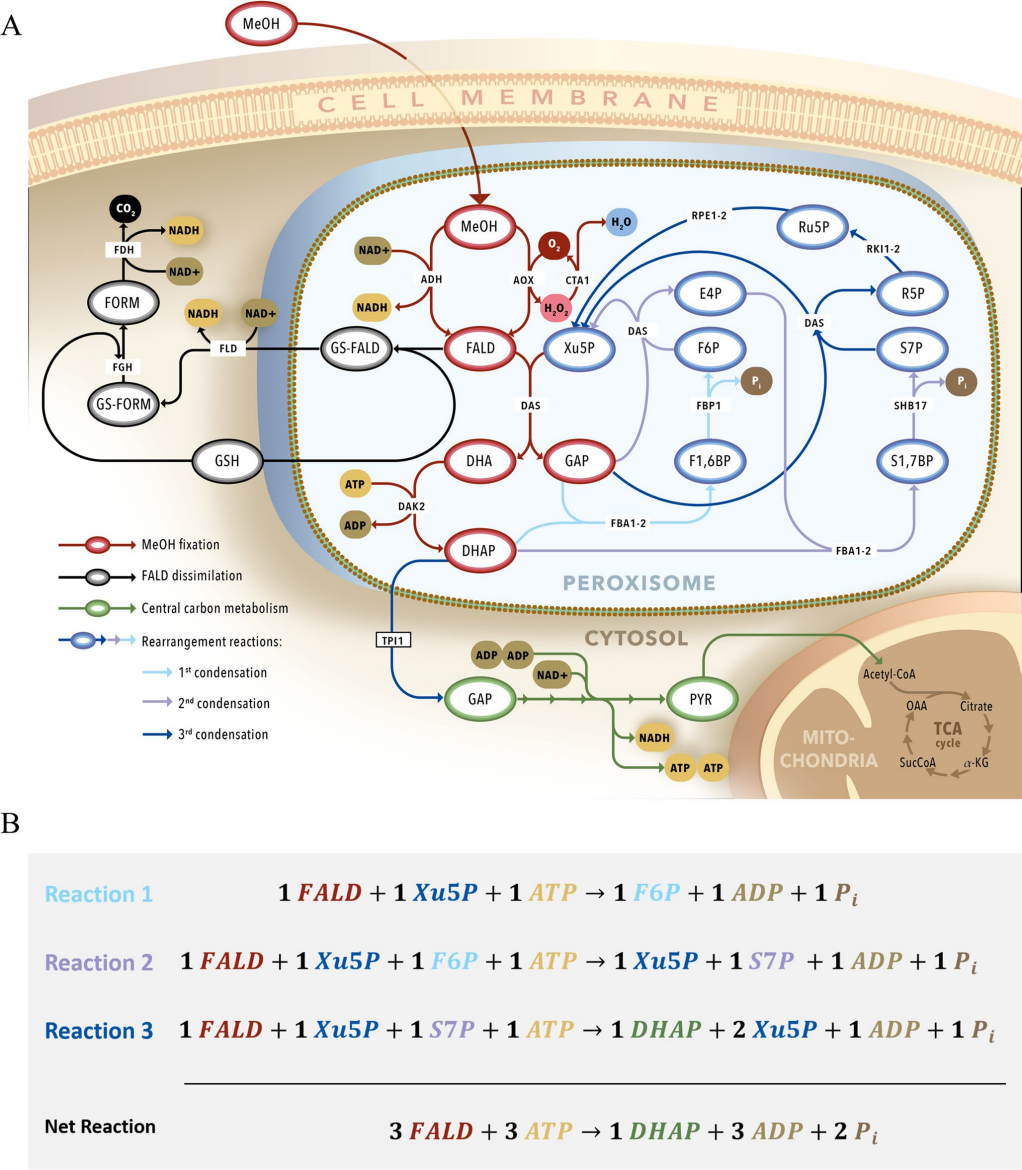
**A **Localization of the Methanol Utilization Pathway (MUT) within subcellular compartments in methylotrophic yeasts. Metabolites: MeOH, methanol; FALD, formaldehyde; GS, glutathione; FORM, formate; Xu5P, xylulose-5-phosphate; DHA, dihydroxyacetone; GAP, glyceraldehyde-3-phosphate; DHAP, dihydroxyacetone-3-phosphate; F1,6BP, fructose-1,6-bisphosphate; F6P, fructose-6-phosphate; E4P, erythrose-4-phosphate; S1,7BP, sedoheptulose,1–7-bisphosphate; S7P, sedoheptulose-7-phosphate; R5P, ribose-5-phosphate; Ru5P, ribulose-5-phosphate; PYR, pyruvate. Enzymes: ADH, Alcohol dehydrogenase; AOX, alcohol oxidase; CTA1, catalase; FLD, formaldehyde dehydrogenase; FGH, S-formylglutathione hydrolase; FDH, formate dehydrogenase; DAS, dihydroxyacetone synthase; DAK2, dihydroxyacetone kinase; FBA1-2, fructose-bisphosphate aldolase; FBP1, fructose-bisphosphatase; SHB17, sedoheptulose-1,7-bisphosphatase; RKI1-2, ribose-5-phosphate ketol-isomerase; RPE1-2, D-ribulose-5-phosphate-3-epimerase; TPI1, triose-phosphate isomerase; ATP, adenosin triphosphate; ADP; adenosin diphosphate; Pi, phosphate. **B **Stoichiometric balance of the condensation reactions for formaldehyde assimilation through the XuMP cycle

Some microorganisms are able to metabolize methanol in spite of its inherent toxicity, an example of methylotrophy. Methylotrophy comprises a series of metabolic pathways through which highly reduced C1 compounds like methane or methanol are assimilated by microorganisms to obtain energy or funnel the carbon molecules into their catabolism (Abou-Zeid and Baghlaf 1983; Large 2012; Wegner 1990; Yurimoto et al. 2011). Bacterial species can utilize a number of these compounds through different metabolic pathways and have more synthetic modules available to increase the energetic efficiencies of their assimilation (Chistoserdova et al. 2009; Kremp and Müller 2021; Krüsemann et al. 2023). Regarding methanol, combinations of different pathways like the ribulose monophosphate (RuMP) or the reductive glycine, and engineered cofactor systems, like NAD- or quinone-dependent, enable an optimisation of methanol assimilation where the reductive power is conserved and better utilized into cell energetics (Krüsemann et al. 2023; Singh et al. 2022; Wagner et al. 2023). Methylotrophic bacteria, however, have a lower resistance to the toxic intermediates generated in the assimilation process and release high amounts of heat as a result of their rapid growth, both traits that are generally undesirable in bioprocesses. Contrarily, methylotrophic yeasts can exclusively process methanol *via* the native peroxisome-localized Methanol Utilization (MUT) Pathway involving the Xylulose Mono-Phosphate (XuMP) cycle (Fig. 2A). This pathway consists of three major steps: the initial conversion of methanol to formaldehyde, its subsequent dissimilation into energy and CO_2_, or its assimilation through C3 intermediates into cell components and further metabolic products. The initial conversion to formaldehyde is typically performed by an alcohol oxidase (AOX), though research has found that, in some species, a promiscuous alcohol dehydrogenase (ADH) can also carry out the conversion in the absence of AOX (Yurimoto et al. 2011; Zavec et al. 2021).

AOX activity relies on O_2_ as an electron acceptor, which is converted to highly reactive hydrogen peroxide (H_2_O_2_). Being a highly oxidizing agent, hydrogen peroxide can pose a dire threat to cellular homeostasis through protein inactivation, membrane disruption or DNA damage (Finnegan et al. 2010; Juven and Pierson 1996). As part of a highly specialized system to deal with this oxidative stress in methylotrophic yeasts, AOX activity is localized within peroxisomes alongside catalase (CAT) to detoxify its H_2_O_2_ by-product into H_2_O and O_2_. (Rußmayer et al. 2015; van der Klei et al. 2006). This localization enables the rapid removal of H_2_O_2_ within the cell by spatially coupling both enzymatic reactions, thereby minimizing the damage done to critical biomolecules and organelles. A second mechanism to reduce oxidative stress within the peroxisome for reactive oxygen species (ROS) scavenging is performed by the glutathione-dependant peroxiredoxin Pmp20 (Bener Aksam et al. 2008; Berrios et al. 2022; Horiguchi et al. 2001). The enzyme reduces hydroperoxides into their non-ROS variants, thus preventing their oxidative capabilities. Mutant yeast cells lacking Pmp20 but expressing CAT have been shown to experience stronger growth impairments than CAT-negative mutants; this emphasises the critical action of the enzyme on mitigating oxidative stress and might point at an underlying activity on general ROS removal (Horiguchi et al. 2001; Yurimoto et al. 2011). Another detoxifying step tackles the non-enzymatical hemiacetal formation of methanol and formaldehyde, which is oxidized to methyl formate by a cytosolic alcohol dehydrogenase (ADH) with methyl formate synthase activity (Murdanoto et al. 1997; Yurimoto et al. 2004).

The produced formaldehyde can then follow a dissimilation route where it is fully oxidized to CO_2_ in the cytosol (Berrios et al. 2022; Yurimoto et al. 2005). It leaves the peroxisome following a non-enzymatic condensation with reduced glutathione and is then subject to two consecutive dehydrogenation reactions to formate and CO_2_ with the co-production of two NADH molecules. A cytosolic system for the regeneration of reduced glutathione is also found in methylotrophic yeast (Yano et al. 2009). Flux Balance Analysis (FBA) studies have shown that a very high percentage (> 50%) of the methanol fed to the cells is dissimilated into CO_2_, especially in protein production processes, where an upregulation of the TCA cycle has also been studied (Jordà et al. 2012; Krüsemann et al. 2023; Leticia Vanz et al. 2012; Singh et al. 2022). Different growth rates and methanol uptake rates, as well as cofeeding strategies also have a direct effect on the amount of methanol directly dissimilated to CO_2_, sometimes rising to values above 90% loss (Jordà et al. 2012, 2014). This showcases a dire weak point in carbon conservation rates in methylotrophic yeasts and reinforces the need to look closely at the choice of feed, feeding rate and cultivation conditions for each individual production application of methylotrophic yeasts.

Contrarily, the assimilation pathway of methanol entails the condensation of formaldehyde with Xylulose-5-Phosphate (Xu5P) through the activity of the dihydroxyacetone synthase (DAS) enzyme, generating one molecule of dihydroxyacetone (DHA) and one molecule of glyceraldehyde-3-phosphate (GAP). DHA is further phosphorylated by the dihydroxyacetone kinase (DAK) into dihydroacetone-3-phosphate (DHAP) with the consumption of one molecule of ATP. These C3 molecules constitute the basis for the rest of the reactions in the assimilation pathway, involving a number of rearrangement reactions through enzymes from the pentose phosphate pathway (PPP). Figure 2A summarizes a series of three condensation reactions of formaldehyde through the PPP reactions proposed by Rußmayer et al. (2015). The net gain of these reactions is one C3 molecule per three molecules of formaldehyde, with the concomitant consumption of three molecules of ATP (Fig. 2B). This C3 molecule can then follow a branching path, where it can be further assimilated catabolically through the lower glycolysis, or anabolically through gluconeogenesis or the PPP.

Contrary to the traditional belief that these rearrangements happened cytosolically through the PPP, (Hartner and Glieder 2006; Yurimoto et al. 2011), Rußmayer et al. (2015) showed that methylotrophic yeasts possess a duplication at genome-level for some of the PPP enzymes that carry an additional C-terminal peroxisomal targeting signal (PTS). They further proved these enzymes were enriched in peroxisomal preparations of *K. phaffi*, thus demonstrating the existence of a compartmentalized cyclic XuMP pathway within the peroxisome separate from the PPP. While not all enzymes predicted to be involved in the cytosolic PPP model have a peroxisomal isoform, other enzymes with similar activities are proposed to compensate for their absence, like could be the case for DAS catalysing other transketolase as the enzyme has been shown to work in vitro with other physiological substrates (Bystrykh et al. 1981; Ro et al. 1997).

The MUT pathway sees its first line of regulation through its localization within one subcellular compartment, the peroxisome. Peroxisome deficient mutants (Cregg et al. 1990; Liu et al. 1992) exhibit strong growth defects or a complete inability to grow when cultivated on methanol as the carbon source, even though they express functionally active versions of the enzymes required for methanol metabolism (Tan et al. 1995; van der Klei et al. 1991). Furthermore, peroxisome biogenesis is induced in the presence of methanol, as demonstrated by their high occurrence in electron-transmission microscopy images of methylotrophic yeast cells grown on methanol (van der Klei et al. 2006; Veenhuis et al. 1983). Transcriptional studies made on *O. polymorpha* (van Zutphen et al. 2010) and *K. phaffi* (Rußmayer et al. 2015) also show that peroxisome related genes (PEX) are upregulated in cells grown on methanol. These genes encode peroxins which are the proteins responsible for the biosynthesis, proliferation and function of peroxisomes. Interestingly, this upregulation of PEX genes was functionally differential, with only the genes and proteins related to the methanol assimilation pathway showing an increased expression when compared to PEX genes that carry other metabolic functions like β-oxidation of fatty acids (Prielhofer et al. 2015). Exemplary, PEX5, receptor of the PTS1 signal, and PTS1-harboring genes have been associated with methanol metabolism and showed to be enriched in methanol-grown yeast cells, in contrast to PEX7, the counterpart receptor for the PTS2 signal peptide associated with fatty acid metabolism (Liang et al. 2012; van Zutphen et al. 2010). Along with the activation of methanol metabolism, the mechanisms to mitigate oxidative stress are also transcriptionally regulated upon ROS and aldehyde production through Yap1p, which upregulates the expression of CAT, Pmp20, and the enzymes part of the glutathione recovery system (Rodrigues-Pousada et al. 2019; Yano et al. 2009).

The choice of carbon source also modulates the MUT pathway. When grown on glucose, the peroxisomal enzymes are significantly downregulated, whereas growth on methanol exerts a strong induction of the pathway (Yurimoto et al. 2011). These effects have been observed in a transcriptomic level for *O. polymorpha* (van Zutphen et al. 2010) and in a transcriptomic and proteomic level for *K. phaffi* (Liang et al. 2012; Prielhofer et al. 2015; Rußmayer et al. 2015). Enzyme levels for AOX and DAS are elevated on methanol-grown cells, but DAS exhibits a faster expression after induction (Sakai et al. 1996), a mechanism thought to be evolved to reduce the toxicity of formaldehyde by enabling its condensation immediately upon production after the start of the expression of AOX (Yurimoto 2009). Beside methanol induction, these genes also experience different expression levels on different carbon sources, with varying degrees of activity depending on the species of yeast (Yurimoto et al. 2011). The change between glucose and methanol as carbon source has been extensively studied and described (Kang and Gellissen 2004; Hartner and Glieder 2006; Kunze et al. 2009; Yurimoto et al. 2000; Yurimoto and Sakai 2019). Methanol genes are repressed by glucose even at very low concentrations, a mechanism attributed to the evolutionary advantage of utilizing more efficient carbon sources (Dusny and Schmid 2016). Upon glucose depletion or exposure to non-fermentable substrates like glycerol, the genes for methanol assimilation experience variable expression levels depending on the species: *C. boidinii* and *O. polymorpha* show around 10% and 80% of the maximum AOX activity, respectively, whereas *K. phaffi* does not exhibit derepression of the genes without methanol exposure (Kunze et al. 2009; Yurimoto et al. 2011). The mechanism for the methanol-specific induction has been described to be mediated through species-specific transcription factors: Trm1/2p and Hap complex in *C. boidinii* (Oda et al. 2015, 2016; Sasano et al. 2008, 2010), Mpp1p in *O. polymorpha* (Leão-Helder et al. 2003; Wang et al. 2016), Mxr1 and Mit1 in *K. phaffi* (Gupta et al. 2021; Lin-Cereghino et al. 2006; Wang et al. 2016). These transcription factors mediate the induction by methanol at two levels: first at a derepression level (Trm2/Mxr1) upon glucose depletion and further at an actual induction level by methanol (Trm1/Mpp1/Hap) (Yurimoto and Sakai 2019).

## Frontiers of metabolic engineering in Methylotrophic yeasts for non-proteinogenic products

Traditional chemical routes to produce bulk and fine chemicals are mostly cost-effective and reliable. However, increasingly stringent regulations on GHG emissions, coupled with concerns over pollution, the depletion of fossil resources, and market volatility, are driving the search for alternatives. In this context, microbial cell factories are promising platforms for contributing to a shift from a linear to a circular economy (Cho et al. 2022). Although microbial systems currently face challenges in terms of economic competitiveness compared to chemical synthesis, they offer several advantages, including safety, scalability, and well-established methodologies (Jozala et al. 2016). A crucial limitation in the advancement of microbial biomanufacturing lies in the availability and cost of feedstocks. This has driven a growing interest in microbial hosts capable of utilising unconventional, low-cost, and sustainable substrates, such as third-generation derived ones, i.e., green methanol.

Methylotrophic yeasts, particularly the genera *Ogataea*, *Komagataella*, *Kuraishia*, and *Candida*, have gained attention for their unique metabolism and industrially favourable traits. These organisms can grow on methanol as their sole carbon and energy source. In addition, they possess tolerance to low pH and a Crabtree-negative phenotype, which prevents the accumulation of toxic by-products, such as ethanol (Hagman et al. 2014). Additionally, they display the ability to perform correct and compatible post-translational modification, e.g., protein folding, glycosylation, disulphide-bond formation, and an efficient secretion system (Cereghino and Cregg 2000; Damasceno et al. 2012; Karbalaei et al. 2020). Lastly, they possess a tight gene regulation and strong induction of methanol-inducible promoters.

Protein production in methylotrophic yeasts is reviewed elsewhere (Tsuda and Nonaka 2024; Stasyk and Stasyk 2025). In this review, we focus on non-proteinogenic products and the metabolic engineering advances in *Ogataea* and *Komagataella*, while *Kuraishia* and *Candida* are not debated here as the research on them is still at early stages.

### Ogataea

The recently classified genus *Ogataea*, formerly known as *Hansenula*, comprises many species known for their role in fermentation and the production of chemicals (Yamada et al. 1994). This success is mainly due to its high tolerance towards high temperatures (growth described up to 50 °C), acidic pH and osmotic stress. Some of the strains of this genus have been fully sequenced, e.g., *Ogataea polymorpha* NCYC495 and DL-1, and their genomes have been assembled into chromosomes. Nevertheless, the whole genome sequence is available as contings only and is not fully annotated (Chang et al. 2022). Promoters, terminators, and plasmids have been widely described and characterised. In *O. polymorpha*, promoters like the methanol oxidase promoter (pMOX), the catalase promoter (pCAT) and the dihydroxyacetone synthase promoter (pDHAS) showcase the highest activities with methanol as a C-source (Fig. 2A) (Zhai et al. 2021). Terminators showed an impact on gene expression, affecting mRNA stability but independently of the C-source (Wefelmeier et al. 2022).

Until recently, genetic and metabolic engineering by precise genome modifications in *Ogataea* was laborious, consisting in flanking gene markers with 500/1000 bp long DNA fragments and homologous recombination (HR) in the genome (González et al. 1999). The non-homologous end-joining (NHEJ) mechanism is the preferred DNA-repair route in *Ogataea*, which leads to random genomic integration and represents a bottleneck for targeted genetic mutations. To overcome the latter, gene *YKU80*, responsible for the NHEJ activity involved in heterodimer formation, was knocked out, increasing HR efficiency (Prasitchoke et al. 2007; Saraya et al. 2012). Recently, genetic engineering possibilities expanded thanks to the establishment of CRISPR-Cas9 systems (Numamoto et al. 2017; Wang et al. 2018; Gao et al. 2021; Juergens et al. 2018).

Many molecules, mainly platform and fine chemicals, have been produced in *Ogataea* using methanol as the sole carbon and energy source (Manfrão-Netto et al. 2019). L-Malic acid production was achieved via expression of reductive tricarboxylic acid (rTCA) cycle in the cytosol of *O. polymorpha* NCYC495 leu1.1 ∆yku80 by integration of three codon-optimised genes under the control of methanol-inducible promoters (Riley et al. 2016). In detail, the pyruvate carboxylase (PYC) under the control of pMOX, the malate dehydrogenase (MDH) controlled by pCAT, both from *Rhizopus oryzae*, and the malic acid transporter (MAE1) from *Schizosaccharomyces pombe* under control of pDHAS. Malate titre reached up to 14 g/L on minimal medium with 1% (v/v) methanol pulses every 24 h (Wefelmeier et al. 2024).

L-lactate production was achieved via metabolic engineering and Adaptive Laboratory Evolution (ALE). Production of L-lactate was achieved in *O. polymorpha* NCYC495 leu1.1 via genome integration of the codon-optimised L-lactate dehydrogenase (LDH) from *Lactobacillus helveticus* in the *HIS2* genomic locus and expression under the control of the pMOX promoter and tMOX terminator. The obtained titre was 3.0 g/L, which was further improved to 3.8 g/L through ALE of the same strain by daily 0.5% v/v pulses (Wefelmeier et al. 2023).

Acetone was produced at a titre of 8.6 mg/L. The genetic engineering approach has been the same as described for malate and lactate. The codon-optimised sequences coding for three enzymes from *Clostridium acetobutylicum* were integrated into the *O. polymorpha* genome under the regulation of methanol-inducible promoters pMOX and pCAT. In order, these are the acetyl-CoA acetyltransferase, the butyrate-acetoacetate CoA-transferase and the acetoacetate decarboxylase. The engineered yeast was supplied with 0.5% (v/v) methanol as the carbon source, and acetate was added at low concentrations to the cultivation to improve production (Wefelmeier et al. 2024).

Isoprene production is another example of an achievable metabolic engineering possibility in *Ogataea.* The gene coding for isoprene synthase Isps(p) from the plant *Populus alba* and the truncated version of the 3-hydroxy-3-methylglutaryl-CoA reductase gene from *Saccharomyces cerevisiae* (N-terminal signal) were integrated into the *O. polymorpha* genome under control of pDHAS and pMOX, respectively. The highest titre reported is 4.4 g/L, feeding 0.5% (v/v) methanol (Wefelmeier et al. 2024).

The production of another major platform chemical, i.e., hydroxypropionate (HP), was also achieved in *O. polymorpha*. Biologically, it is synthesised from the cellular central intermediate malonyl-CoA. A mutated version (N940V, K1106W and S1114R) of malonyl‐CoA reductase (MCR) from *Chloroflexus aurantiacus* with improved catalytic efficiency for 3‐HP production and under the control of the methanol-induced promoter pAOX was introduced in *O. polymorpha* genome. Together with precursor and cofactor enhancement and a fed a titre of 7.10 g/L was achieved (Liu et al. 2016; Yu et al. 2023).

Largely used in the detergent and cosmetic industries, fatty alcohols were produced by Zhai et al. (2023) from methanol *via* a multisided approach and reached a titre of 3.6 g/L. First, the fatty alcohols synthetic pathway was segregated into the peroxisomes by expressing the fatty acyl-CoA reductase (*TaFAR1*) from *Tyto alba*, commonly known as barn owl, and the alcohol dehydrogenase (*ScADH5)* from *S. cerevisiae*. Additionally, *O. polymorpha* ZX-F75U was genetically engineered sequentially to optimise the supply of peroxisomal fatty acyl-CoA and NADPH by (i) overexpressing in the peroxisomes the endogenous *PYC1*, *MDH3* and the heterologous *ME1* from *Rhodosporidium toruloides*, (ii) enhance cell robustness by deleting a putative lipase (*LPL1*) and a membrane protein related to zinc metabolism *(IZH3)*, and (iii) enhance methanol tolerance by overexpressing the dihydroxyacetone synthase gene *DAS2* (Zhai et al. 2023).

With its complex 15-carbon cycle structure molecule, its high cetane number and sustainable nature, β-farnesene is a peculiar specialty chemical produced in *O. polymorpha*. Its production from methanol was achieved with extensive metabolic engineering in the fatty acid overproducing strain M16. Specifically, the geranyl/farnesyl diphosphate synthase-encoding gene (*ERG20*) was fused with the β-farnesene synthase-encoding gene (*FS*) from the plant *Artemisia annua* and expressed under the control of the methanol-inducible promoter pTAL1. Native truncated 3-hydroxy-3-methylglutaryl coenzyme A reductase (*tHMGR)* and *HMGR* from *Silicibacter pomeroyi*, fused *ERG10-ERG13*,* ERG12*,* ERG8*, and *ERG19* were overexpressed. Last, the β-oxidation pathway was systematically overexpressed, and the cytosolic malate synthase (*MLS1*) was knocked out (Li et al. 2024).

### Komagataella

The methylotrophic yeast belonging to the *Komagataella* genus, classified until 1995 as *Pichia pastoris* (Yamada et al. 1995) and renamed today as *Komagataella phaffii*, is a well-established platform for recombinant protein production (Wu et al. 2023). The *K. phaffii* genome has been fully sequenced and annotated (De Schutter et al. 2009), and its success in research and industry is due to its ability to grow to high-cell densities on minimal media, being Crabtree negative (Cereghino and Cregg 2000) and displaying high-production yields. Notably, genetic tools for *K. phaffii* have been widely described and developed. In 1985, the gene coding for alcohol oxidase (AOX1), the enzyme involved in the first enzymatic step of the methanol oxidation pathway, and its promoter (pAOX1) were characterised (Cregg et al. 1985; Ellis et al. 1985). Afterwards, expression vectors, integrative vectors, and selection markers were described (Cereghino and Cregg 2000). Like in other methylotrophic yeasts, the recombination machinery in *K. phaffii* is less efficient than in *S. cerevisiae*. Therefore, a functional CRISPR-Cas9 system for *K. phaffii* was recently developed (Weninger et al. 2016; Gao et al. 2022; Wu et al. 2023).

Based on old and new technologies, significant progress has been made to expand the product array in *K. phaffii*, using methanol as the sole carbon and energy source. Guo et al. (2021) reported malate production using a multisided approach. First, a methanol-inducible rTCA pathway from *Rhizopus oryzae* was expressed. Genomic integration was achieved via integrative plasmids bearing heterologous genes, pyruvate carboxylase (PYC) and malate dehydrogenase (MDH) under the control of the pAOX1 promoter. To overcome a common bottleneck in malic acid production, the malate transporter (MAE) from *S. pombe* was integrated into the genome and overexpressed under the control of pAOX1. In addition, to redirect metabolic fluxes from the XuMP cycle (Fig. 2A), glucose-6-phosphate isomerase was deleted. Lastly, the cultivation medium was supplemented with 1 g/L yeast extract as an additional nitrogen source. This multisided approach produced 2.79 g/L of malic acid from methanol (Guo et al. 2021a).

Another organic acid of industrial interest, D-lactic acid, was produced from methanol at a final titre of 3.48 g/L. *K. phaffii* does not synthesise lactate natively. Therefore, the gene coding for lactate dehydrogenase (LDH) from *Leuconostoc mesenteroides* was integrated into the genome, under the control of native pAOX1 and tAOX1 terminator. The integration was achieved using a multi-copy integrative vector targeting the non-transcribed spacer (NTS) of the ribosomal DNA (rDNA) locus, yielding a high gene copy number (Yamada et al. 2019). Recently, Bachleitner et al. (2024) produced 17 g/L of lactate from methanol in a bioreactor, using an engineered *K. phaffii* strain. This was modified by (a) integration in the *RGI2* locus of the codon-optimised L-lactate dehydrogenase from *Lactiplantibacillus plantarum* under the control of pAOX1 and (b) deletion of the L-lactate cytochrome-c oxidoreductase *CYB2* using the CRISPR-Cas9 system. Even though this integration was enough to produce lactate, it was only via overexpression of transcriptional activators *MXR1* and *MIT1* that the titre increased significantly (Bachleitner et al. 2024). To produce itaconic acid, Severinsen et al. (2024) engineered *Komagataella phaffii* via integration of the heterologous genes from *Aspergillus terreus* for the itaconate metabolic pathway. Specifically, the gene responsible for the conversion of cis-aconitate to itaconate, cadA, was integrated into the yeast genome under the control of pAOX1. The transporters’ genes mttA and mfsA were integrated into the genome under the control of a moderately strong and strong constitutive promoter, respectively. Further process optimisation, i.e., increase of temperature to 32 °C and adoption of a multiphase process, in a bioreactor led to titres of 55 g/L after circa 5 days of methanol feed.

Another interesting example showcasing the possibilities of using methanol as a fermentation substrate is β-alanine, a non-essential amino acid, and the only natural β-amino acid. This molecule is a precursor of many nitrogen-based compounds, but its industrial synthesis requires harsh, not environmentally friendly conditions. In *K. phaffii*, 5.6 g/L of β-alanine was produced by introducing copies of the L-aspartate-α-decarboxylase (*ADC*) from *Bacillus subtilis* and the aspartate dehydrogenase (*AspDH*) from *Serratia proteamaculans* encoding genes in its genome, using integration vectors. Both genes were placed under the control of the methanol-inducible promoter pAOX1 (Miao et al. 2021). Two different groups were able to produce 3-hydroxypropionate (3-HP) from methanol in *K. phaffii* (Àvila-Cabré et al. 2023; Chen et al. 2025).

Two different groups independently achieved a notable 3-HP titre in this yeast. In detail, Chen et al. (2025) were able to produce 23 g/L of 3-HP following the heterologous expression of two genes from the β-alanine synthetic pathway, *AspDH* from *Brucella anthropic* and *ADC* from *Bacillus subtilis*, in one and three copies respectively integrated into the genome. Moreover, to optimise the metabolic flux of pyruvate and avoid the formation of acetic acid, the aldehyde dehydrogenase gene (*ALD4*) was knocked-out. Lastly, the final titre was additionally increased after the integration of the expression cassette constructed fusing the β-alanine pyruvate aminotransferase gene (*BAPAT*) from *Bacillus cereus* under the pAOX promoter and the malonate semialdehyde reductase gene (*MCRN*) under the PS2 promoter. On the other hand, Àvila-Cabré et al. (2023) were able to reach a titre of 21.4 g/L of 3-HP. Here, the β-alanine pathway was constructed via integration into *K. phaffii* genome of two copies of *panD* under the control of pAOX1-from *Tribolium castaneum* (2x), *BAPAT* from *Bacillus cereus* under the control of pFDH1, and *ydfG* from *Escherichia coli* K-12 under the moderately strong promoter pPOR.

Fatty alcohols are part of the plethora of chemicals produced via genetic engineering of *K. phaffii*. Shen et al. (2024) engineered and optimised the cytosolic fatty alcohol biosynthesis in the yeast by integrating into its genome the alcohol dehydrogenase gene (*ScADH5*) from *S. cerevisiae* under the control of pTEF1 and knocking-out the aldehyde dehydrogenase gene (*hfd1*). Then the codon optimized fatty acyl-CoA reductases (*FAR*) from *Mus musculus* was selected and heterologously expressed under the control of pTEF1 promoter and targeted to the peroxisome via a signal peptide. In this strain, the methanol assimilation pathway was also strengthened, by overexpressing DAS2 under its natural promoter, with good results. Noteworthy, is also the intervention, i.e., overexpression under the control of pPGI1 of *PEX8*, on the peroxisomes, aiming at coupling methanol assimilation and fatty alcohols production. The highest titre was only obtained when the cytoplasmic and peroxisomal engineering were combined in the diploid *K. phaffii* SYW15 strain, reaching a titre of 5.6 g/L (Shen et al. 2024). Several other bulk, fine chemicals and pharmaceuticals were synthesised in *K. phaffii* using methanol as the sole carbon source, although in low amounts, and are listed in Table 2.

**Table 2:** Alphabetical list of bulk-, fine-, and specialty chemicals produced from methanol in the two most prominent methylotrophic yeast, *Ogataea* and *Komagataella*

Microorganism	Product	Category	Titre	Genetic engineering strategy	Yield gP/gMeOH	Productivity	References
*Ogataea*	Acetone	Bulk	8.6 mg/L	Heterologous expression under methanol-inducible promoters of pCAT-*THLA* from *C. acetobutylicum*, pMOX-*YbgC* from *H. influenzae*, and pMOX-Adc from *P. polymyxa*	n.q.	n.q.	Yu et al. (2023), Wefelmeier et al. (2023), Wefelmeier et al. (2024), Zhai et al. (2023), Li et al. (2024)
	Fatty alcohols	Bulk	3.6 g/L	Heterologous expression of *TaFAR1* from *T. alba* and *ADH5* from *S. cerevisiae.* Overexpression of *IDP2* from *S. cerevisiae* and deletion of *LPL1*, *IZH3*. Overexpression of endogenous *PYC1* and *MDH3*, and *RtME1*. Overexpression of *DAS2*	0.016	12.9 mg/L/h	
	Hydroxypropionate	Fine	7.10 g/L	Heterologous expression under methanol-inducible promoter: pAOX-*MCR* from *C. aurantiacus*	n.q.	n.q.	
	Isoprene	Bulk	4.4 g/L	Heterologous expression under methanol-inducible promoters: pDHAS-Isps(p) from *P. alba* and pMOX-hydroxy-3-methylglutaryl-CoA reductase from *S. cerevisiae*	n.q.	n.q.	
	L-Lactic acid	Bulk	3.8 g/L	Heterologous expression under methanol-inducible promoter and terminator: pMOX-LDH-tMOX from *L. helveticus*, and ALE	0.02	12.6 mg/L/h	
	Malic acid	Fine	13 g/L	Heterologous rTCA expression under methanol-inducible promoters: pMOX-PYC, pCAT-MDH, both from *R. oryzae*, pDHAS-MAE1 from *S. pombae*	0.41	3.3 g/L/day	
	β-farnesene	Specialty	14.7 g/L	Heterologous expression of pTAL1-*ERG20* fused with *FS* from *A. annua* and overexpression of *tHMGR* and *HMGR* from *S. pomeroyi*, fused *ERG10* and *ERG13* (2x), *ERG12*, *ERG8* and *ERG19.* Overexpression of β-oxidation pathway and knock out of *MLS1*	0.046	48 mg/L/h	
*Komagataella*	Astaxanthin	Specialty	716.13 mg/L	Heterologous expression of *CrtE*, *CrtYB*, *CrtI*, *CrtYB*, *CrtZ*, *CrtW*	n.q.	n.q.	Yamada et al. (2019), Miao et al. (2021), Guo et al. (2021), Gao et al. (2022), Bachleitner et al. (2024), Severinsen et al. (2024), Shen et al. (2024), Chen et al. (2025), Àvila-Cabré et al. (2023), Wang et al. (2025)
	Chondroitin sulphate	Specialty	2.1 g/L	Heterologous expression of *KFOA* and *KFOC* from *E. coli* expressing *TUAD* from *B. subtilis.* Overexpression of *ATPS* and *APSK.*	n.q.	n.q.	
	Citrinin	Specialty	0.6 mg/L	Heterologous expression of PKSCT, MPL1, MPL2, and MPL4 from *M. purpureus*, MPL6 and MPL7 from *M. ruber*, and NPGA from *A. nidulans*	n.q.	n.q.	
	D-Lactic acid	Bulk	3.48 g/L	Heterologous expression of pAOX1-LDH-tAOX1 from *L. mesenteroides*	0.22	36.3 ± 2.0 mg/L/h	
	Fatty alcohols	Bulk	5.6 g/L	Combination of (i) heterologous expression of pTEF1-*ADH5* from *S. cerevisiae* and pTEF1-*FAR* from *M. musculus*, (ii) overexpression of pDAS-*DAS2* and pPGI1-*PEX8*, (iii) knock-out of *hfd1* in *K. phaffii* SYW15.	0.13	n.q.	
	3-Hydroxypropionate	Bulk	23 g/L 21.4 g/L	Heterologous expression of *AspDH* from *B. anthropic* and *ADC from B. subtilis* (x3). ALD4 deletion; pAOX-*BAPAT–PS*_*2*_-*McrN* fusion cassette expression with both genes from *B. cereus* Heterologous expression of pPOR1-*panD* from *T. castaneum* (2x), *yhxA* from *B. cereus*, and *ydfG* from *E. coli K-12*	0.12 0.15	n.q. 0.48 g/L/h	
	Hyaluronic acid	Specialty	1.7 g/L	Heterologous expression of *xhasa*2 and *xhasb* from *X. laevis*. Overexpression of *HASC*, *HASD*, and *HASE*	n.q.	n.q.	
	Itaconic acid	Bulk	55 g/L	Heterologous expression and overexpression of pAOX1-*cadA*, pPOR1-*mttA* and pGAP-*mfsA* from *A. terreus*	0.24	0.45 g/L/h	
	L-Lactic acid	Bulk	17 g/L	Heterologous expression of pAOX1-*LDH* from *L. plantarus*; deletion of Nrg1, Mig1-1, and Mig1-2; overexpression of transcriptional activators Mxr1 and Mit1	n.q.	n.q.	
	Lovastatin	Specialty	250.8 mg/L	Heterologous expression of *LovB*, *LovC*, and *LovG* from *A. terreus* and *NpgA* from *A. nidulans*	n.q.	n.q.	
	Lycopene	Specialty	714 mg/L	Heterologous expression of CrtB, CrtI, and CrtE from *C. glutamicum*, overexpression of *hmgr*, and *hmgs*. Increase of *ggpps* copy number	n.q.	n.q.	
	Malic acid	Fine	2.79 g/L	Heterologous rTCA expression under methanol-inducible promoters: PYC, MDH both from *R. oryzae*, MAE1 from *S. pombae*. Deletion of Ku70, ODC1p, PDC, GPI	n.q.	n.q.	
	Monacolin J	Specialty	593.9 mg/L	Expression of *LovA* and split of CPR pathway	n.q.	n.q.	
	Zeaxanthin	Specialty	n.q.	Heterologous expression of *CrtI*, *CrtE-CrtZ*, *CrtYB*	n.q.	n.q.	
	2,3-butanediol	Bulk	2.2 g/L	Heterologous expression of *AlsS*, *AlsD*, *BDH*,	n.q.	n.q.	
	6-methyl salicylic acid	Fine	2.2 g/L	Heterologous coexpression of NpgA from *A. nidulans* and *atX* from *A. terrus*	n.q.	n.q.	
	α-bisabolene	Specialty	270 mg/L	Heterologous expression of pTEF1-tHMGR and pGCW14-BIS fused with FPPS from *A. grandis* (multiple copies). Overexpression of *ERG10* and *HMGS* under pTEF1. Overexpression of MK and PMK.	0.01	2.25 mg/L/h	
	β-alanine	Fine	5.6 g/L	Heterologous expression of PAOX1-ADC, SpeAspDH (overexpressed) from *B. subtilis*, deletion of Ku70	n.q.	n.q.	
	β-carotene	Specialty	n.q.	Heterologous expression of *CrtE*, *CrtYB*, *CrtI*, *CrtYB*, *CrtZ*	n.q.	n.q.	
	β-farnesene	Specialty	338 mg/L	Heterologous expression of pGCW14-*FS* from *A. annua* fused with FPPS	0.02	2.8 mg mg/L/h	

Titre, genetic engineering strategy, yield in grams of product/grams of methanol and productivity are listed; (n.q. = non-quantified)

## Process considerations in methanol fermentation

Compared to other C1 feedstocks, e.g., methane, the nature and characteristics of methanol offer several benefits as a substrate in industrial processes. Methanol is a colourless, odourless, and water-miscible liquid. High water solubility is especially crucial to avoid gradients in nutrient concentration and mass transfer limitations that could otherwise impair fermentation efficiency (Crater and Lievense 2018). Noteworthy is methanol’s flexibility as a feedstock: deriving from fossil or renewable sources, it allows transitioning from one to the other as the technology develops and market prices or policies shift. Lastly, its transport, manipulation, and storage are convenient, being a liquid at room temperature (Olah 2005). Nevertheless, handling methanol needs careful consideration. The molecule is toxic and possesses high volatility and flammability. A major concern is the risk of explosion due to methanol vapour accumulation, which can easily occur in industrial settings. For these reasons, industrial facilities handling methanol must comply with regulations that vary by country. Typically, large-scale fermentation facilities must have explosion-proof equipment, safe storage, constant and continuous monitoring of methanol concentration, and proper ventilation (Antoniewicz 2019; Gan et al. 2023), increasing capital expenditure (CAPEX).

The remarkable flexibility and benefits of methanol-based processes also derive from the microorganisms employed. Naturally, microbial fermentations and bioconversions do not require extreme working conditions, e.g., high pressure, high temperature, harsh chemicals, or catalysts. This makes them not only sustainable but also cost-effective and safer. Additionally, the availability of methanol-inducible promoters in methylotrophic yeasts, such as the alcohol oxidase gene or the catalase gene promoters, allows precise gene transcription activation and, hence, efficient fine-tuning of metabolic pathway activation (Yan et al. 2022). In addition, they possess tight regulation and noteworthy strength (Cregg et al. 1993; Wu et al. 2023). Consequently, different fermentation approaches have been developed and optimised; indeed, multiple-phase fermentation is the most suitable when using methanol as the induction substrate for methanol-induced product production (Gan et al. 2023). First, it allows the decoupling of the growth and production phases. Since both are highly energetically and metabolically demanding, this increases final biomass density and production efficiency. Secondly, it concedes precise induction of genes controlled by methanol-induced promoters, preventing intermediates toxicity of methanol metabolism, i.e. formaldehyde, and generating higher final titres and yields (Ahmad et al. 2014; Tsuda and Nonaka 2024).

**Fig. 3:**
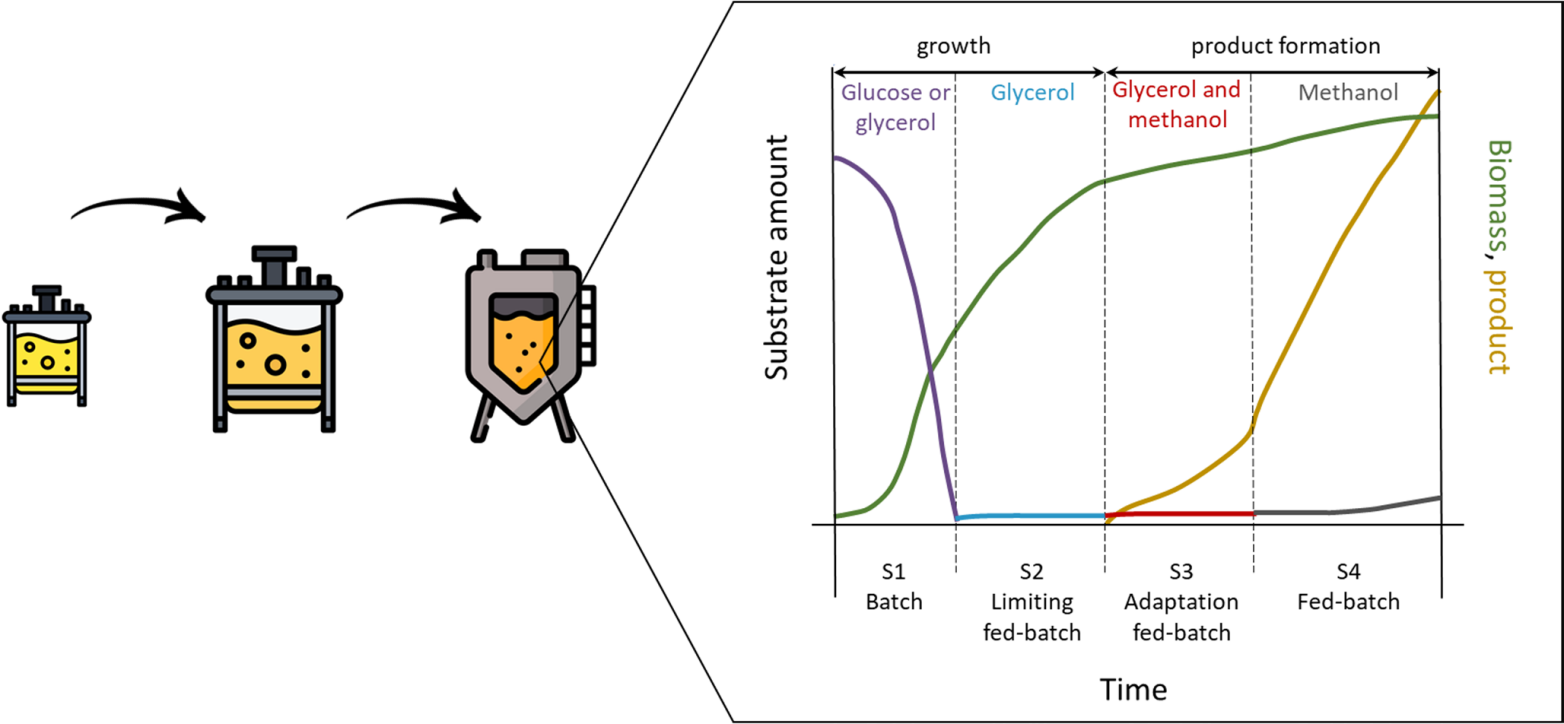
Graphical description of a generic fermentation process using a methylotrophic yeast. Aseed fermentation on complex medium is followed by a batch and fed-batch phase whereglucose, glycerol and methanol are the main feedstocks and the production of the targetcompound is triggered by methanol addition

In this context, a typical multi-phase process (Fig. 3) begins with the first phase (S1), designed as a batch phase to grow biomass with either glucose or glycerol, respectively. Once the set amount of biomass is achieved, the second phase (S2) focuses on production, i.e., a fed-batch phase with methanol as source of carbon and energy is initiated (Brierley 1998; Potvin et al. 2012). A three-phase process has also been developed, where the S1 and S3 phase follow the previously described approach but a new intermediate phase (S2) is introduced: a fed-batch phase, which uses glycerol to further increase cell density, like in S1 (Liu et al. 2016; Potvin et al. 2012; Severinsen et al. 2024; Totaro et al. 2021). In the latter S2, the feeding rate can be either constant or follow an exponential feeding profile (Potvin et al. 2012). To have a smoother transition from S2 and S3, methanol and glycerol can be co-fed during an additional phase for a given length of time, during which glycerol concentration in the feed gradually decreases, while the concentration of methanol increases accordingly (Cereghino and Cregg 2000; Zhang et al. 2000).

In the *Ogataea* genus, glycerol can derepress the methanol oxidase promoter up to 80% compared to the activation on methanol only. Hence, the transition phase can have a meaningful impact on the final production in *Ogataea*. Lastly, the fed-batch phase (S3) using methanol can be further optimised by tuning it on the microorganism’s methanol uptake rate and the goal of the process. This can be done by adopting different feeding strategies: constant Dissolved Oxygen feeding (DO-stat), constant specific growth rate feeding, constant methanol concentration feeding, oxygen-limited fed-batch (OLFB), and temperature-limited fed-batch (TLFB) (Potvin et al. 2012). During the entire duration of the process, methanol level monitoring is essential. The substrate concentration must always be kept within a well-defined range, typically between 0.5% v/v and 2.5% v/v. In fact, too high concentrations will hinder growth and toxify the cultivation broth. This is mainly due to formaldehyde accumulation which will lead to cell death; on the other hand, too low concentrations can slow down the production (Wang et al. 2023). Despite these process-control-intensive constraints, using methanol ensures a minimal risk of contamination, an amenable condition in industrial settings where maintaining sterility is challenging (Cregg et al. 2000).

An important economical consideration, particularly in large-scale processes, is the oxygen transfer efficiency (OTE) and the heat release associated with methanol metabolism (Singh et al. 2022). Methanol fermentation, as a highly oxidative process, is oxygen-demanding due to the initial reaction in methylotrophic yeast metabolism and the necessity to re-oxidise the NADH pool generated by the assimilation and product pathways (Fig. 2A). At the metabolic level, pathways with higher carbon efficiency, i.e., those directing a greater proportion of methanol carbon into product rather than CO₂, also exhibit markedly lower O₂ demand, since less substrate oxidation is needed for redox balancing (Wagner et al. 2023). This relationship is particularly relevant in high-cell-density fermentations (HCDF), where insufficient OTE leads to methanol accumulation and incomplete substrate-to-product conversion, reducing final titres and yields in oxygen-limited conditions (Liu et al. 2016; Mate et al. 2013; Shen et al. 2022). To compensate, pure oxygen supplementation is required during the process, which significantly raises Operating Expenditure. Alternatively, process intensification strategies such as super-atmospheric aeration can reduce oxygen-related costs by 20–30% across multiple production cycles by eliminating the need for pure oxygen (Campani et al. 2016). Liu et al. (2016) demonstrated an effective approach to overcome oxygenation limitation in a HCDF without the need of pure oxygen addition. The group increased the reactor air pressure from 0.05 MPa (super atmospheric pressure) to 0.10 ± 0.05 MPa to enhance gas solubility. Although specialised equipment was required, the feasibility of the removal of a pure oxygen supplement was successfully demonstrated increasing the volumetric production by 43% and reducing costs (Charoenrat et al. 2006; Liu et al. 2016).

## Conclusion and outlook

In this review, we showcased how methylotrophic yeasts have emerged as promising microbial platforms for converting methanol into value-added specialty and fine chemicals. Within the framework of a circular economy, methanol as a substrate is particularly attractive: it can be derived sustainably from 3G feedstocks, i.e., captured CO₂ and renewable energy; and its use in biotechnology will benefit from existing demands of the chemical industry. Moreover, industrial infrastructure for methanol-based chemical production is in place and at scale, thereby minimising CAPEX and facilitating a smooth transition from fossil-based to renewable sources. Hence, “land-free” methanol offers an advantageous “drop-in” solution that reduces investment efforts during the shift to more sustainable feedstocks for chemical production processes.

To favour and fulfil the ambitious goal of reshaping industrial production and achieve the predicted bioeconomy scenario, intensive technology development and maturity are especially urgent. In fact, the biotechnological conversion of methanol *via* yeast fermentation has still some major limitations that hinder economic feasibility and attractiveness. This is especially accurate for bulk chemicals production, that are usually produced via well-stablished and efficient chemical processes. Chemical catalysis can achieve carbon conversion efficiencies around and above 90% and volumetric productivities of tons of product per m³ per day. In contrast, bioconversion requires a certain degree of complexity and generally exhibits conversion efficiencies rarely above 60%, along with low volumetric productivities. Considering methanol high degree of reduction, inherently lower conversion yields, influenced by the lower reduction state of the product, are to be expected. Furthermore, the volumetric productivity in a fermentation process is generally lower of several orders of magnitude compared to a catalytic process. Protein production remains the most established and economically viable application. Therefore, bioprocess development should first focus on high-value, low-volume products, i.e., fine and specialty chemicals, that are not easily synthesised via chemical routes. This strategy could accelerate the establishment of C1 feedstocks in the sector, which would boost and expand the technology maturity to then develop processes for other chemicals in a lower price range.

While considerable challenges remain, particularly regarding process economics and strain performance, targeted innovations in strain engineering, promoter design, and upstream feedstock sourcing could position methylotrophic yeasts as competitive and sustainable platforms for the next generation of bio-based manufacturing.

## Data Availability

No datasets were generated or analysed during the current study.

## References

[CR1] Abou-Zeid A-ZA, Baghlaf AO (1983) Methanol as the carbon source of production of single-cell proteins (SCP-s). Zentralbl Mikrobiol 138(6):451–464. 10.1016/S0232-4393(83)80044-4

[CR2] Ahmad M, Hirz M, Pichler H, Schwab H (2014) Protein expression in *Pichia pastoris*: recent achievements and perspectives for heterologous protein production. Appl Microbiol Biotechnol 98(12):5301–5317. 10.1007/s00253-014-5732-5 24743983 10.1007/s00253-014-5732-5 PMC4047484

[CR3] Antoniewicz MR (2019) Synthetic methylotrophy: strategies to assimilate methanol for growth and chemicals production. Curr Opin Biotechnol 59:165–174. 10.1016/j.copbio.2019.07.001 31437746 10.1016/j.copbio.2019.07.001

[CR4] Araújo TP, Mitchell S, Pérez-Ramírez J (2024) Design principles of catalytic materials for CO_2_ hydrogenation to methanol. Adv Mater 36(48):2409322. 10.1002/adma.202409322 39300859 10.1002/adma.202409322 PMC11602685

[CR5] Àvila-Cabré S, Pérez-Trujillo M, Albiol J (2023) Engineering the synthetic β-alanine pathway in *Komagataella phaffii* for conversion of methanol into 3-hydroxypropionic acid. Microb Cell Fact 22:237. 10.1186/s12934-023-02241-9 37978380 10.1186/s12934-023-02241-9 PMC10655335

[CR6] Bachleitner S, Severinsen MM, Lutz G et al (2024) Overexpression of the transcriptional activators Mxr1 and Mit1 enhances lactic acid production on methanol in *Komagataella phaffii*. Metab Eng 85:133–144. 10.1016/j.ymben.2024.07.013 39067842 10.1016/j.ymben.2024.07.013

[CR7] Bener Aksam E, Jungwirth H, Kohlwein SD (2008) Absence of the peroxiredoxin Pmp20 causes peroxisomal protein leakage and necrotic cell death. Free Radic Biol Med 45(8):1115–1124. 10.1016/J.FREERADBIOMED.2008.07.010 18694816 10.1016/j.freeradbiomed.2008.07.010

[CR8] Bennett RK, Gregory GJ, Gonzalez JE, et al (2021) Improving the methanol tolerance of an *Escherichia coli* methylotroph via adaptive laboratory evolution enhances synthetic methanol utilization. Front Microbiol 12:638426. 10.3389/FMICB.2021.638426/BIBTEX 10.3389/fmicb.2021.638426 PMC7904680 33643274

[CR9] Bernal-Cabas M, Kumar K, Terpstra O, et al (2025) Food production from air: gas precision fermentation with hydrogen-oxidising bacteria. Trends Biotechnol. 10.1016/j.tibtech.2025.08.003 10.1016/j.tibtech.2025.08.003 40930898

[CR10] Berrios J, Theron CW, Steels S et al (2022) Role of dissimilative pathway of *Komagataella phaffii* (*Pichia pastoris*): formaldehyde toxicity and energy metabolism. Microorganisms. 10.3390/MICROORGANISMS10071466 35889185 10.3390/microorganisms10071466 PMC9321669

[CR11] Brierley RA (1998) Secretion of recombinant human insulin-like growth factor I (IGF-I). In: Higgins DR, Cregg JM (eds), Pichia Protocols. Humana Press, pp 149–177. 10.1385/0-89603-421-6:149 10.1385/0-89603-421-6:149 9680639

[CR12] Bystrykh LV, Sokolov AP, Trotsenko YA (1981) Purification and properties of dihydroxyacetone synthase from the methylotrophic yeast *Candida boidinii*. FEBS Lett 132(2):324–328. 10.1016/0014-5793(81)81189-1

[CR13] Campani G, Possedente dos Santos M, Gonçalves da Silva G et al (2016) Recombinant protein production by engineered *Escherichia coli* in a pressurized airlift bioreactor: A techno-economic analysis. Chem Eng Process: Process Intensif 103:63–69. 10.1016/j.cep.2015.10.020

[CR14] Cereghino JL, Cregg JM (2000) Heterologous protein expression in the methylotrophic yeast *Pichia pastoris*. FEMS Microbiol Rev 24(1):45–66. 10.1111/j.1574-6976.2000.tb00532.x 10640598 10.1111/j.1574-6976.2000.tb00532.x

[CR15] Cereghino GPL, Cereghino JL, Ilgen C et al (2002) Production of recombinant proteins in fermenter cultures of the yeast *Pichia pastoris*. Curr Opin Biotechnol 13(4):329–332. 10.1016/S0958-1669(02)00330-0 12323354 10.1016/s0958-1669(02)00330-0

[CR16] Chang J, Bei J, Shao Q, Wang H et al (2022) Full-length genome of an *Ogataea polymorpha* strain CBS4732 ura3∆ reveals large duplicated segments in subtelomeric regions. Front Microbiol 13:855666. 10.3389/fmicb.2022.855666 35464988 10.3389/fmicb.2022.855666 PMC9019687

[CR17] Charoenrat T, Ketudat-Cairns M, Jahic M et al (2006) Increased total air pressure versus oxygen limitation for enhanced oxygen transfer and product formation in a *Pichia pastoris* recombinant protein process. Biochem Eng J 30(2):205–211. 10.1016/j.bej.2006.04.004

[CR18] Chen NH, Djoko KY, Veyrier FJ et al (2016) Formaldehyde stress responses in bacterial pathogens. Front Microbiol 7:257. 10.3389/FMICB.2016.00257 26973631 10.3389/fmicb.2016.00257 PMC4776306

[CR19] Chen S, Zhang M, Wu X et al (2025) Promoter engineering for enhanced 3-hydroxypropionic acid production in *Pichia pastoris*. Synth Syst Biotechnol 10(3):916–924. 10.1016/j.synbio.2025.04.013 40421287 10.1016/j.synbio.2025.04.013 PMC12104163

[CR20] Chistoserdova L, Kalyuzhnaya MG, Lidstrom ME (2009) The expanding world of methylotrophic metabolism. Annu Rev Microbiol 63:477–499. 10.1146/annurev.micro.091208.073600 19514844 10.1146/annurev.micro.091208.073600 PMC2827926

[CR21] Cho JS, Kim GB, Eun H, Moon CW, Lee SY (2022) Designing microbial cell factories for the production of chemicals. JACS Au 2(8):1781–1799. 10.1021/jacsau.2c00344 10.1021/jacsau.2c00344 PMC9400054 36032533

[CR22] Crater JS, Lievense JC (2018) Scale-up of industrial microbial processes. FEMS Microbiol Lett 365(13):fny138. 10.1093/femsle/fny138 29860483 10.1093/femsle/fny138 PMC5995164

[CR23] Cregg JM, Barringer KJ, Hessler AY et al (1985) *Pichia pastoris* as a host system for transformations. Mol Cell Biol 5(12):3376–3385. 10.1128/mcb.5.12.3376-3385.1985 3915774 10.1128/mcb.5.12.3376-3385.1985 PMC369166

[CR24] Cregg JM, Van Klei IJ, Sulter GJ et al (1990) Peroxisome-deficient mutants of *Hansenula polymorpha*. Yeast 6(2):87–97. 10.1002/YEA.320060202

[CR25] Cregg JM, Cereghino JL, Shi J et al (2000) Recombinant protein expression in *Pichia pastoris*. Mol Biotechnol 16(1):23–52. 10.1385/MB:16:1:23 11098467 10.1385/MB:16:1:23

[CR26] Cregg JM, Vedvick TS, Raschke WC (1993) Recent advances in the expression of foreign genes in *Pichia pastoris*. Bio/Technology 11(8):905–910. 10.1038/nbt0893-905 7763913 10.1038/nbt0893-905

[CR27] Damasceno LM, Huang C-J, Batt CA (2012) Protein secretion in *Pichia pastoris* and advances in protein production. Appl Microbiol Biotechnol 93(1):31–39. 10.1007/s00253-011-3654-z 22057543 10.1007/s00253-011-3654-z

[CR28] Daniell J, Köpke M, Simpson SD (2012) Commercial biomass syngas fermentation. Energies 5(12):5372–5417. 10.3390/en5125372

[CR29] De Schutter K, Lin Y-C, Tiels P et al (2009) Genome sequence of the recombinant protein production host *Pichia pastoris*. Nat Biotechnol 27(6):561–566. 10.1038/nbt.1544 19465926 10.1038/nbt.1544

[CR30] Du L, Su Y, Sun D et al (2008) Formic acid induces Yca1p-independent apoptosis-like cell death in the yeast *Saccharomyces cerevisiae*. FEMS Yeast Res 8(4):531–539. 10.1111/J.1567-1364.2008.00375.X 18452540 10.1111/j.1567-1364.2008.00375.x

[CR31] Dusny C, Schmid A (2016) The MOX promoter in *Hansenula polymorpha* is ultrasensitive to glucose-mediated carbon catabolite repression. FEMS Yeast Res 16(6):67. 10.1093/FEMSYR/FOW067 10.1093/femsyr/fow067 27527102

[CR32] Ellis SB, Brust PF, Koutz PJ et al (1985) Isolation of alcohol oxidase and two other methanol regulatable genes from the yeast *Pichia pastoris*. Mol Cell Biol 5(5):1111–1121 3889590 10.1128/mcb.5.5.1111-1121.1985 PMC366829

[CR33] Finnegan M, Linley E, Denyer SP (2010) Mode of action of hydrogen peroxide and other oxidizing agents: differences between liquid and gas forms. J Antimicrob Chemother 65(10):2108–2115. 10.1093/JAC/DKQ308 20713407 10.1093/jac/dkq308

[CR34] Gan Y, Meng X, Gao C et al (2023) Metabolic engineering strategies for microbial utilization of methanol. Engineering Microbiology 3(3):100081. 10.1016/j.engmic.2023.100081 39628934 10.1016/j.engmic.2023.100081 PMC11611044

[CR35] Gao J, Gao N, Zhai X et al (2021) Recombination machinery engineering for precise genome editing in methylotrophic yeast *Ogataea polymorpha*. iScience 24(3):102168. 10.1016/j.isci.2021.102168 33665582 10.1016/j.isci.2021.102168 PMC7907465

[CR36] Gao J, Xu J, Zuo Y et al (2022) Synthetic biology toolkit for marker-less integration of multigene pathways into *Pichia pastoris* via CRISPR/Cas9. ACS Synth Biol 11(2):623–633. 10.1021/acssynbio.1c00307 35080853 10.1021/acssynbio.1c00307

[CR37] Garcia JA, Villen-Guzman M, Rodriguez-Maroto JM (2022) Technical analysis of CO_2_ capture pathways and technologies. J Environ Chem Eng 10(5):108470. 10.1016/j.jece.2022.108470

[CR38] Neha GP, Upadhyay SN, Dubey SK (2020) Bio-methanol as a renewable fuel from waste biomass: Current trends and future perspective. Fuel 273:117783. 10.1016/j.fuel.2020.117783

[CR39] Global hydrogen review (2022) IEA. https://www.iea.org/reports/global-hydrogen-review-2022

[CR40] González C, Perdomo G, Tejera P et al (1999) One-step, PCR-mediated, gene disruption in the yeast *Hansenula polymorpha*. Yeast 15(13):1323–1329. 10.1002/(SICI)1097-0061(19990930)15:13%3C;1323::AID-YEA459%3E;3.0.CO;2-1 10509014 10.1002/(SICI)1097-0061(19990930)15:13<1323::AID-YEA459>3.0.CO;2-1

[CR41] Guo F, Dai Z, Peng W et al (2021) Metabolic engineering of *Pichia pastoris* for malic acid production from methanol. Biotechnol Bioeng 118(1):357–371. 10.1002/bit.27575 32965690 10.1002/bit.27575

[CR42] Gupta A, Rao KK, Sahu U et al (2021) Characterization of the transactivation and nuclear localization functions of *Pichia pastoris* zinc finger transcription factor Mxr1p. J Biol Chem 297(4):101247. 10.1016/J.JBC.2021.101247 34582889 10.1016/j.jbc.2021.101247 PMC8526985

[CR43] Hagman A, Säll T, Piškur J (2014) Analysis of the yeast short-term Crabtree effect and its origin. FEBS J 281(21):4805–4814. 10.1111/febs.13019 10.1111/febs.13019 PMC4240471 25161062

[CR44] Hartner FS, Glieder A (2006) Regulation of methanol utilisation pathway genes in yeasts. Microb Cell Fact 5:39. 10.1186/1475-2859-5-39 10.1186/1475-2859-5-39 PMC1781073 17169150

[CR45] Higgins DR, Cregg JM (1998) Introduction to *Pichia pastoris*. In: Higgins DR, Cregg JM (eds), *Pichia* Protocols. Humana Press, pp 1–15. 10.1385/0-89603-421-6:1 10.1385/0-89603-421-6:1 9680629

[CR46] Horiguchi, H., Yurimoto, H., Kato, N., et al. (2001). Antioxidant system within yeast peroxisome: biochemical and physiological characterization of CbPmp20 in the methylotrophic yeast *Candida boidinii*. J Biol Chem 276(17):14279–14288. 10.1074/JBC.M011661200 10.1074/jbc.M011661200 11278957

[CR47] IRENA, & Methanol Institute (2021) Innovation outlook: renewable methanol. International Renewable Energy Agency

[CR48] Jayakody LN, Jin YS (2021) In-depth understanding of molecular mechanisms of aldehyde toxicity to engineer robust *Saccharomyces cerevisiae*. Appl Microbiol Biotechnol 105(7):2675–2692. 10.1007/S00253-021-11213-1 33743026 10.1007/s00253-021-11213-1

[CR49] Jordà J, De Jesus SS, Peltier S (2014) Metabolic flux analysis of recombinant *Pichia pastoris* growing on different glycerol/methanol mixtures by iterative fitting of NMR-derived 13 C-labelling data from proteinogenic amino acids. New Biotechnol 31(1):120–132. 10.1016/j.nbt.2013.06.007 10.1016/j.nbt.2013.06.007 23845285

[CR50] Jordà J, Jouhten P, Cámara E et al (2012) Metabolic flux profiling of recombinant protein secreting *Pichia pastoris* growing on glucose:methanol mixtures. Microb Cell Fact 11:57. 10.1186/1475-2859-11-57 22569166 10.1186/1475-2859-11-57 PMC3443025

[CR51] Jozala AF, Geraldes DC, Tundisi LL, de Feitosa VA, Breyer CA, Cardoso SL, Mazzola PG, de Oliveira-Nascimento L, de Rangel-Yagui CO, de Magalhães PO, de Oliveira MA, Pessoa A (2016) Biopharmaceuticals from microorganisms: From production to purification. Braz. J Microbiol 47(Suppl 1):51–63. 10.1016/j.bjm.2016.10.007 27838289 10.1016/j.bjm.2016.10.007 PMC5156500

[CR52] Juergens H, Varela JA, Gorter de Vries, et al (2018) Genome editing in *Kluyveromyces* and *Ogataea* yeasts using a broad-host-range Cas9/gRNA co-expression plasmid. FEMS Yeast Res 18(3):foy012. 10.1093/femsyr/foy012 10.1093/femsyr/foy012 PMC6018904 29438517

[CR53] Juven BJ, Pierson MD (1996) Antibacterial effects of hydrogen peroxide and methods for its detection and quantitation. J Food Prot 59(11):1233–1241. 10.4315/0362-028X-59.11.1233 31195444 10.4315/0362-028X-59.11.1233

[CR54] Kang HA, Gellissen G (2004) *Hansenula polymorpha*. In: Production of Recombinant Proteins. Wiley, pp 111–142. 10.1002/3527603670.ch6

[CR55] Kang S, Boshell F, Goeppert A, et al (2021) Innovation outlook: renewable methanol. In: Gielen D, Dolan G (eds). International Renewable Energy Agency

[CR56] Karbalaei M, Rezaee SA, Farsiani H (2020) *Pichia pastoris*: a highly successful expression system for optimal synthesis of heterologous proteins. J Cell Physiol 235(9):5867–5881. 10.1002/jcp.29583 32057111 10.1002/jcp.29583 PMC7228273

[CR57] Kassem II, Candelero-Rueda RA, Esseili KA et al (2017) Formate simultaneously reduces oxidase activity and enhances respiration in *Campylobacter jejuni*. Sci Rep 7(1):1–11. 10.1038/SREP40117 28091524 10.1038/srep40117 PMC5238407

[CR58] Keleman A, Rañó HG (2012) The Mexican tortilla crisis of 2007: The impacts of grain-price increases on food-production chains. In: Global Food-Price Shocks and Poor People. Routledge

[CR59] Kremp F, Müller V (2021) Methanol and methyl group conversion in acetogenic bacteria: biochemistry, physiology and application. FEMS Microbiol Rev 45(2):1–22. 10.1093/FEMSRE/FUAA040 10.1093/femsre/fuaa040 32901799

[CR60] Krüsemann JL, Rainaldi V, Cotton CA et al (2023) The cofactor challenge in synthetic methylotrophy: bioengineering and industrial applications. Curr Opin Biotechnol 82:102953. 10.1016/J.COPBIO.2023.102953 37320962 10.1016/j.copbio.2023.102953

[CR61] Kunze G, Kang HA, Gellissen G (2009) *Hansenula polymorpha* (*Pichia angusta*): biology and applications. Yeast biotechnology: diversity and applications, pp 47–64. 10.1007/978-1-4020-8292-4_3

[CR62] Lais A, Gondal M, Dastageer M, Al Adel F (2018) Experimental parameters affecting the photocatalytic reduction performance of CO_2_ to methanol: a review. Int J Energy Res 42. 10.1002/er.3965

[CR63] Large P (2012) Methylotrophy and methanogenesis. Springer

[CR64] Leão-Helder AN, Krikken AM, Van der Klei IJ et al (2003) Transcriptional down-regulation of peroxisome numbers affects selective peroxisome degradation in *Hansenula polymorpha*. J Biol Chem 278(42):40749–40756. 10.1074/JBC.M304029200 12902346 10.1074/jbc.M304029200

[CR65] Lee S-E, Park B-S, Yoon J-J (2010) Proteomic evaluation of cellular responses of *Saccharomyces cerevisiae* to formic acid stress. Mycobiology 38(4):302. 10.4489/MYCO.2010.38.4.302 23956670 10.4489/MYCO.2010.38.4.302 PMC3741523

[CR66] Leticia Vanz A, Lünsdorf H, Adnan A, et al (2012) Physiological response of *Pichia pastoris* GS115 to methanol-induced high level production of the Hepatitis B surface antigen: catabolic adaptation, stress responses, and autophagic processes. 10.1186/1475-2859-11-103 10.1186/1475-2859-11-103 PMC3539919 22873405

[CR67] Li J, Gao J, Ye M et al (2024) Engineering yeast for high-level production of β-farnesene from sole methanol. Metab Eng 85:194–200. 10.1016/j.ymben.2024.08.006 39181436 10.1016/j.ymben.2024.08.006

[CR68] Liang S, Wang B, Pan L, et al (2012) Comprehensive structural annotation of *Pichia pastoris* transcriptome and the response to various carbon sources using deep paired-end RNA sequencing. BMC Genomics 13(1):1–14. 10.1186/1471-2164-13-738/TABLES/2 10.1186/1471-2164-13-738 PMC3547764 23276294

[CR69] Lin-Cereghino GP, Godfrey L, Cruz BJdela et al (2006) Mxr1p, a key regulator of the methanol utilization pathway and peroxisomal genes in *Pichia pastoris*. Mol Cell Biol 26(3):883–897. 10.1128/MCB.26.3.883-897.2006 16428444 10.1128/MCB.26.3.883-897.2006 PMC1347016

[CR70] Liu H, Tan X, Veenhuis M et al (1992) An efficient screen for peroxisome-deficient mutants of *Pichia pastoris*. J Bacteriol 174(15):4943–4951. 10.1128/JB.174.15.4943-4951.1992 1629154 10.1128/jb.174.15.4943-4951.1992 PMC206307

[CR71] Liu W-C, Gong T, Wang Q-H (2016) Scaling-up fermentation of *Pichia pastoris* to demonstration-scale using new methanol-feeding strategy and increased air pressure instead of pure oxygen supplement. Sci Rep. 10.1038/srep18439 26790977 10.1038/srep18439 PMC4726300

[CR72] Liu X, Park H, Ackermann YS et al (2025) Exploring biotechnology for plastic recycling, degradation and upcycling for a sustainable future. Biotechnol Adv 81:108544. 10.1016/j.biotechadv.2025.108544 40024585 10.1016/j.biotechadv.2025.108544

[CR73] Liu Z, Wang K, Chen Y (2020) Third-generation biorefineries as the means to produce fuels and chemicals from CO_2_. Nat Catal 3(3):274–288. 10.1038/s41929-019-0421-5

[CR74] Liu Z, Shi S, Ji Y et al (2023) Opportunities of CO_2_-based biorefineries for production of fuels and chemicals. Green Carbon 1(1):75–84. 10.1016/j.greenca.2023.09.002

[CR75] Liu C, Ding Y, Zhang R et al (2016) Functional balance between enzymes in malonyl-CoA pathway for 3-hydroxypropionate biosynthesis. Metab Eng 34:104–111. 10.1016/j.ymben.2016.01.001 26791242 10.1016/j.ymben.2016.01.001

[CR76] Macauley-Patrick S, Fazenda ML, McNeil B et al (2005) Heterologous protein production using the *Pichia pastoris* expression system. Yeast 22(4):249–270. 10.1002/yea.1208 15704221 10.1002/yea.1208

[CR77] Madejski P, Chmiel K, Subramanian N et al (2022) Methods and techniques for CO_2_ capture: review of potential solutions and applications in modern energy technologies. Energies 15(3):3. 10.3390/en15030887

[CR78] Manfrão-Netto JHC, Gomes AMV, Parachin NS (2019) Advances in using *Hansenula polymorpha* as chassis for recombinant protein production. Front Bioeng Biotechnol. 10.3389/fbioe.2019.00094 31119131 10.3389/fbioe.2019.00094 PMC6504786

[CR79] Mate DM, Gonzalez-Perez D, Kittl R et al (2013) Functional expression of a blood tolerant laccase in *Pichia pastoris*. BMC Biotechnol 13:38. 10.1186/1472-6750-13-38 23627343 10.1186/1472-6750-13-38 PMC3655043

[CR80] Miao L, Li Y, Zhu T (2021) Metabolic engineering of methylotrophic *Pichia pastoris* for the production of β-alanine. Bioresour Bioprocess 8(1):89. 10.1186/s40643-021-00444-9 38650288 10.1186/s40643-021-00444-9 PMC10991944

[CR81] Monlau F, Sambusiti C, Barakat A et al (2012) Predictive models of biohydrogen and biomethane production based on the compositional and structural features of lignocellulosic materials. Environ Sci Technol 46(21):12217–12225. 10.1021/es303132t 23050634 10.1021/es303132t

[CR82] Murdanoto AP, Sakai Y, Konishi T et al (1997) Purification and properties of methyl formate synthase, a mitochondrial alcohol dehydrogenase, participating in formaldehyde oxidation in methylotrophic yeasts. Appl Environ Microbiol 63(5):1715–1720. 10.1128/AEM.63.5.1715-1720.1997 9143107 10.1128/aem.63.5.1715-1720.1997 PMC168467

[CR83] NASA, Global Climate Change (2025) Carbon dioxide concentration. Climate.Nasa.Gov. https://climate.nasa.gov/vital-signs/carbon-dioxide?intent=121

[CR84] Numamoto M, Maekawa H, Kaneko Y (2017) Efficient genome editing by CRISPR/Cas9 with a tRNA-sgRNA fusion in the methylotrophic yeast *Ogataea polymorpha*. J Biosci Bioeng 124(5):487–492. 10.1016/j.jbiosc.2017.06.001 28666889 10.1016/j.jbiosc.2017.06.001

[CR85] Oakley JL, Bicknell JE (2022) The impacts of tropical agriculture on biodiversity: a meta-analysis. J Appl Ecol 59(12):3072–3082. 10.1111/1365-2664.14303

[CR86] Oda S, Yurimoto H, Nitta N (2015) Molecular characterization of hap complex components responsible for methanol-inducible gene expression in the methylotrophic yeast *Candida boidinii*. Eukaryot Cell 14(3):278–285. 10.1128/EC.00285-14 25595445 10.1128/EC.00285-14 PMC4346567

[CR87] Oda S, Yurimoto H, Nitta N (2016) Unique c-terminal region of Hap3 is required for methanol-regulated gene expression in the methylotrophic yeast *Candida boidinii*. Microbiology 162(5):898–907. 10.1099/MIC.0.000275 26963751 10.1099/mic.0.000275

[CR88] Ogata K, Ohsugi M, Nishikawa H (1969) A yeast capable of utilizing methanol. Agric Biol Chem 33(10):1519–1520. 10.1271/BBB1961.33.1519

[CR89] Olah GA (2005) Beyond oil and gas: the methanol economy. Angew Chem Int Ed Engl 44(18):2636–2639. 10.1002/anie.200462121 15800867 10.1002/anie.200462121

[CR90] Panich J, Fong B, Singer SW (2021) Metabolic engineering of *Cupriavidus necator* H16 for sustainable biofuels from CO_2_. Trends Biotechnol 39(4):412–424. 10.1016/j.tibtech.2021.01.001 33518389 10.1016/j.tibtech.2021.01.001

[CR91] Potvin G, Ahmad A, Zhang Z (2012) Bioprocess engineering aspects of heterologous protein production in *Pichia pastoris*: a review. Biochem Eng J 64:91–105. 10.1016/j.bej.2010.07.017

[CR92] Prasitchoke P, Kaneko Y, Sugiyama M et al (2007) Functional analysis of very long-chain fatty acid elongase gene, HpELO2, in the methylotrophic yeast *Hansenula polymorpha*. Appl Microbiol Biotechnol 76(2):417–427. 10.1007/s00253-007-1012-y 17520249 10.1007/s00253-007-1012-y

[CR93] Prielhofer R, Cartwright SP, Graf AB et al (2015) *Pichia pastoris* regulates its gene-specific response to different carbon sources at the transcriptional, rather than the translational, level. BMC Genomics 16(1):1–17. 10.1186/S12864-015-1393-8/TABLES/6 10.1186/s12864-015-1393-8 PMC4408588 25887254

[CR94] Riley R, Haridas S, Wolfe KH et al (2016) Comparative genomics of biotechnologically important yeasts. Proc Natl Acad Sci U S A 113(35):9882–9887. 10.1073/pnas.1603941113 27535936 10.1073/pnas.1603941113 PMC5024638

[CR95] Ro YT, Eom CY, Song T (1997) Dihydroxyacetone synthase from a methanol-utilizing carboxydobacterium, *Acinetobacter* sp. strain JC1 DSM 3803. J Bacteriol 179(19):6041–6047. 10.1128/JB.179.19.6041-6047.1997 9324250 10.1128/jb.179.19.6041-6047.1997 PMC179506

[CR96] Rodrigues-Pousada C, Devaux F, Caetano SM et al (2019) Yeast AP-1 like transcription factors (Yap) and stress response: a current overview. Microb Cell 6(6):267. 10.15698/MIC2019.06.679 31172012 10.15698/mic2019.06.679 PMC6545440

[CR97] Roy S, Cherevotan A, Peter SC (2018) Thermochemical CO_2_ hydrogenation to single carbon products: scientific and technological challenges. ACS Energy Lett 3(8):1938–1966. 10.1021/acsenergylett.8b00740

[CR98] Rußmayer H, Buchetics M, Gruber C et al (2015) Systems-level organization of yeast methylotrophic lifestyle. BMC Biol 13(1):80. 10.1186/S12915-015-0186-5 26400155 10.1186/s12915-015-0186-5 PMC4580311

[CR99] Sakai Y, Saiganji A, Yurimoto H (1996) The absence of Pmp47, a putative yeast peroxisomal transporter, causes a defect in transport and folding of a specific matrix enzyme. J Cell Biol 134(1):37–51. 10.1083/JCB.134.1.37 8698821 10.1083/jcb.134.1.37 PMC2120916

[CR100] Saraya R, Krikken AM, Kiel JAKW (2012) Novel genetic tools for *Hansenula polymorpha*. FEMS Yeast Res 12(3):271–278. 10.1111/j.1567-1364.2011.00772.x 22129301 10.1111/j.1567-1364.2011.00772.x

[CR101] Sasano Y, Yurimoto H, Yanaka M (2008) Trm1p, a Zn(II)2Cys6-type transcription factor, is a master regulator of methanol-specific gene activation in the methylotrophic yeast *Candida boidinii*. Eukaryot Cell 7(3):527–536. 10.1128/EC.00403-07 18203863 10.1128/EC.00403-07 PMC2268522

[CR102] Sasano Y, Yurimoto H, Kuriyama M et al (2010) Trm2p-dependent derepression is essential for methanol-specific gene activation in the methylotrophic yeast *Candida boidinii*. FEMS Yeast Res 10(5):535–544. 10.1111/J.1567-1364.2010.00640.X 10.1111/j.1567-1364.2010.00640.x 20491943

[CR103] Severinsen MM, Bachleitner S, Modenese V et al (2024) Efficient production of itaconic acid from the single carbon substrate methanol with engineered *Komagataella phaffii*. 10.1101/2024.04.25.591069 10.1186/s13068-024-02541-1 PMC11251334 39010147

[CR104] Shanmugam S, Sekar M, Sivaramakrishnan R et al (2021) Pretreatment of second and third generation feedstock for enhanced biohythane production: challenges, recent trends and perspectives. Int J Hydrogen Energy 46(20):11252–11268. 10.1016/j.ijhydene.2020.12.083

[CR105] Shen D, He X, Weng P et al (2022) A review of yeast: high cell-density culture, molecular mechanisms of stress response and tolerance during fermentation. FEMS Yeast Res 22(1):foac050. 10.1093/femsyr/foac050 36288242 10.1093/femsyr/foac050

[CR106] Shen Y, Cai P, Gao L et al (2024) Engineering high production of fatty alcohols from methanol by constructing coordinated dual biosynthetic pathways. Bioresour Technol 412:131396. 10.1016/j.biortech.2024.131396 39216706 10.1016/j.biortech.2024.131396

[CR107] Si B-C, Li J-M, Zhu Z-B et al (2016) Continuous production of biohythane from hydrothermal liquefied cornstalk biomass via two-stage high-rate anaerobic reactors. Biotechnol Biofuels 9(1):254. 10.1186/s13068-016-0666-z 27895708 10.1186/s13068-016-0666-z PMC5117538

[CR108] Singh HB, Kang M-K, Kwon M et al (2022) Developing methylotrophic microbial platforms for a methanol-based bioindustry. Front Bioeng Biotechnol. 10.3389/fbioe.2022.1050740 36507257 10.3389/fbioe.2022.1050740 PMC9727194

[CR109] Söhngen NL (1905). Methane as carbon-food and source of energy for bacteria (Beijerinck M.W., Trans.). www.dwc.knaw.nl

[CR110] Srinivasan S (2009) The food v. fuel debate: a nuanced view of incentive structures. Renew Energy 34(4):950–954. 10.1016/j.renene.2008.08.015

[CR111] Stasyk O, Stasyk O (2025) Production of recombinant proteins in the methylotrophic yeasts. In Sibirny AA (ed), Biotechnology of yeasts and filamentous fungi. Springer Nature Switzerland, pp 291–320. 10.1007/978-3-031-74726-7_10

[CR112] Tan X, Titorenko VI, van der Klei IJ, et al. (1995) Characterization of peroxisome-deficient mutants of *Hansenula polymorpha*. Curr Genet 28(3):248–257. 10.1007/BF00309784/METRICS 10.1007/BF00309784 8529271

[CR113] Tiso T, Narancic T, Wei R (2021) Towards bio-upcycling of polyethylene terephthalate. Metab Eng 66:167–178. 10.1016/j.ymben.2021.03.011 33865980 10.1016/j.ymben.2021.03.011

[CR114] Totaro D, Radoman B, Schmelzer B, et al (2021) Microscale perfusion-based cultivation for *Pichia pastoris* clone screening enables accelerated and optimized recombinant protein production processes. Biotechnol J 16(3):e2000215. 10.1002/biot.202000215 10.1002/biot.202000215 32935449

[CR115] Tsuda M, Nonaka K (2024) Recent progress on heterologous protein production in methylotrophic yeast systems. World J Microbiol Biotechnol 40(7):200. 10.1007/s11274-024-04008-9 38730212 10.1007/s11274-024-04008-9 PMC11087369

[CR116] Ullmann L, Phan ANT, Kaplan DKP, Blank LM (2021) *Ustilaginaceae* biocatalyst for co-metabolism of CO_2_-derived substrates toward carbon-neutral itaconate production. J Fungi 7(2):98. 10.3390/jof7020098 10.3390/jof7020098 PMC7911105 33573033

[CR117] van Zutphen T, Baerends RJS, Susanna KA et al (2010) Adaptation of *Hansenula polymorpha* to methanol: A transcriptome analysis. BMC Genomics 11(1):1–12. 10.1186/1471-2164-11-1/FIGURES/5 10.1186/1471-2164-11-1 PMC2827406 20044946

[CR118] van der Klei IJ, Harder W, Veenhuis M (1991) Methanol metabolism in a peroxisome-deficient mutant of *Hansenula polymorpha*: a physiological study. Arch Microbiol 156(1):15–23. 10.1007/BF00418181/METRICS 10.1007/BF00418181 1772343

[CR119] van der Klei IJ, Yurimoto H, Sakai Y (2006) The significance of peroxisomes in methanol metabolism in methylotrophic yeast. Biochimica et Biophysica Acta (BBA) - Molecular Cell Research 1763(12):1453–1462. 10.1016/J.BBAMCR.2006.07.016 17023065 10.1016/j.bbamcr.2006.07.016

[CR120] Veenhuis M, Douma A, Harder W, et al (1983) Degradation and turnover of peroxisomes in the yeast *Hansenula polymorpha* induced by selective inactivation of peroxisomal enzymes. Arch Microbiol 134(3):193–203. 10.1007/BF00407757/METRICS 10.1007/BF00407757 6351780

[CR121] Wagner N, Wen L, Frazão CJR et al (2023) Next-generation feedstocks methanol and ethylene glycol and their potential in industrial biotechnology. Biotechnol Adv 69:108276. 10.1016/j.biotechadv.2023.108276 37918546 10.1016/j.biotechadv.2023.108276

[CR122] Wang J, Ma W, Ma W et al (2025) Microbial astaxanthin synthesis by *Komagataella phaffii* through metabolic and fermentation engineering. J Agric Food Chem 73(3):1952–1964. 10.1021/acs.jafc.4c10113 39788928 10.1021/acs.jafc.4c10113

[CR123] Wang L, Deng A, Zhang Y et al (2018) Efficient CRISPR–Cas9 mediated multiplex genome editing in yeasts. Biotechnol Biofuels 11(1):277. 10.1186/s13068-018-1271-0 30337956 10.1186/s13068-018-1271-0 PMC6180501

[CR124] Wang S, Wang Y, Yuan Q (2023) Development of high methanol-tolerance *Pichia pastoris* based on iterative adaptive laboratory evolution. Green Chem 25(21):8845–8857. 10.1039/D3GC02874G

[CR125] Wang X, Wang Q, Wang J et al (2016) Mit1 transcription factor mediates methanol signaling and regulates the alcohol oxidase 1 (AOX1) promoter in *Pichia pastoris*. J Biol Chem 291(12):6245–6261. 10.1074/JBC.M115.692053 26828066 10.1074/jbc.M115.692053 PMC4813576

[CR126] Wang Y, Fan L, Tuyishime P et al (2020) Adaptive laboratory evolution enhances methanol tolerance and conversion in engineered *Corynebacterium glutamicum*. Commun Biol 3(1):1–15. 10.1038/S42003-020-0954-9 32382107 10.1038/s42003-020-0954-9 PMC7205612

[CR127] Wani SR, Jain V (2024) Deciphering the molecular mechanism and regulation of formaldehyde detoxification in *Mycobacterium smegmatis*. Appl Environ Microbiol. 10.1128/AEM.02039-23 38259108 10.1128/aem.02039-23 PMC10880627

[CR128] Wefelmeier K, Schmitz S, Haut AM et al (2023) Engineering the methylotrophic yeast *Ogataea polymorpha* for lactate production from methanol. Front Bioeng Biotechnol 11:1223726. 10.3389/FBIOE.2023.1223726 37456718 10.3389/fbioe.2023.1223726 PMC10347679

[CR129] Wefelmeier K, Schmitz S, Kösters BJ et al (2024) Methanol bioconversion into C3, C4, and C5 platform chemicals by the yeast *Ogataea polymorpha*. Microb Cell Fact 23:8. 10.1186/s12934-023-02283-z 38172830 10.1186/s12934-023-02283-z PMC10763331

[CR130] Wefelmeier K, Ebert BE, Blank LM et al (2022) Mix and match: promoters and terminators for tuning gene expression in the methylotrophic yeast *Ogataea polymorpha*. Front Bioeng Biotechnol 10:876316. 10.3389/FBIOE.2022.876316 35620471 10.3389/fbioe.2022.876316 PMC9127203

[CR131] Wegner GH (1990). Emerging applications of the methylotrophic yeasts. FEMS Microbiol Rev 7(3–4):279–283. 10.1111/J.1574-6968.1990.TB04925.X 10.1111/j.1574-6968.1990.tb04925.x 2094288

[CR132] Welsing G, Wolter B, Kleinert GEK et al (2025) Two-step biocatalytic conversion of post-consumer polyethylene terephthalate into value-added products facilitated by genetic and bioprocess engineering. Bioresour Technol 417:131837. 10.1016/j.biortech.2024.131837 39557102 10.1016/j.biortech.2024.131837

[CR133] Weninger A, Hatzl A-M, Schmid C et al (2016) Combinatorial optimization of CRISPR/Cas9 expression enables precision genome engineering in the methylotrophic yeast *Pichia pastoris*. J Biotechnol 235:139–149. 10.1016/j.jbiotec.2016.03.027 27015975 10.1016/j.jbiotec.2016.03.027

[CR134] Wesner A, Kampe P, Herrmann N et al (2023) Indium-based catalysts for CO_2_ hydrogenation to methanol: key aspects for catalytic performance. ChemCatChem 15(24):e202301125. 10.1002/cctc.202301125

[CR135] Wu X, Cai P, Yao L et al (2023) Genetic tools for metabolic engineering of *Pichia pastoris*. Eng Microbiol 3(4):100094. 10.1016/j.engmic.2023.100094 39628915 10.1016/j.engmic.2023.100094 PMC11611016

[CR136] Yamada R, Ogura K, Kimoto Y et al (2019) Toward the construction of a technology platform for chemicals production from methanol: D-lactic acid production from methanol by an engineered yeast *Pichia pastoris*. World J Microbiol Biotechnol 35(2):37. 10.1007/s11274-019-2610-4 30715602 10.1007/s11274-019-2610-4

[CR137] Yamada Y, Maeda K, Mikata K (1994) The phylogenetic relationships of the hat-shaped ascospore-forming, nitrate-assimilating *Pichia* species, formerly classified in the genus *Hansenula* Sydow et Sydow, based on the partial sequences of 18S and 26S ribosomal RNAs (*Saccharomycetaceae*e): the proposals of three new genera, *Ogataea*, *Kuraishia*, and *Nakazawaea*. Biosci Biotechnol Biochem 58(7):1245–1257. 10.1271/bbb.58.1245 7765249 10.1271/bbb.58.1245

[CR138] Yamada Y, Matsuda M, Maeda K et al (1995) The phylogenetic relationships of methanol-assimilating yeasts based on the partial sequences of 18S and 26S ribosomal RNAs: the proposal of *Komagataella* gen. nov. (*Saccharomycetaceae*). Biosci Biotechnol Biochem 59(3):439–444. 10.1271/bbb.59.439 7766181 10.1271/bbb.59.439

[CR139] Yan C, Yu W, Yao L et al (2022) Expanding the promoter toolbox for metabolic engineering of methylotrophic yeasts. Appl Microbiol Biotechnol 106(9):3449–3464. 10.1007/s00253-022-11948-5 35538374 10.1007/s00253-022-11948-5

[CR140] Yano T, Takigami E, Yurimoto H et al (2009) Yap1-regulated glutathione redox system curtails accumulation of formaldehyde and reactive oxygen species in methanol metabolism of *Pichia pastoris*. Eukaryot Cell 8(4):540. 10.1128/EC.00007-09 19252120 10.1128/EC.00007-09 PMC2669188

[CR141] Ye R-P, Ding J, Gong W et al (2019) CO_2_ hydrogenation to high-value products via heterogeneous catalysis. Nat Commun 10(1):5698. 10.1038/s41467-019-13638-9 31836709 10.1038/s41467-019-13638-9 PMC6910949

[CR142] Yu R, Lai Y, Hartwell HJ (2015) Formation, accumulation, and hydrolysis of endogenous and exogenous formaldehyde-induced DNA damage. Toxicol Sci 146(1):170–182. 10.1093/TOXSCI/KFV079 25904104 10.1093/toxsci/kfv079 PMC4476463

[CR143] Yu W, Gao J, Yao L et al (2023) Bioconversion of methanol to 3-hydroxypropionate by engineering *Ogataea polymorpha*. Chin J Catal 46:84–90. 10.1016/S1872-2067(22)64195-0

[CR144] Yurimoto H, Sakai Y (2019) Methylotrophic yeasts: current understanding of their c1-metabolism and its regulation by sensing methanol for survival on plant leaves. Curr Issues Mol Biol 33:197–209. 10.21775/CIMB.033.197 10.21775/cimb.033.197 31166193

[CR145] Yurimoto H, Oku M, Sakai Y (2011) Yeast methylotrophy: metabolism, gene regulation and peroxisome homeostasis. Int J Microbiol 2011:101298. 10.1155/2011/101298 21754936 10.1155/2011/101298 PMC3132611

[CR146] Yurimoto H, Lee B, Yasuda F et al (2004) Alcohol dehydrogenases that catalyse methyl formate synthesis participate in formaldehyde detoxification in the methylotrophic yeast *Candida boidinii*. Yeast 21(4):341–350. 10.1002/YEA.1101 15042594 10.1002/yea.1101

[CR147] Yurimoto H, Kato N, Sakai Y (2005) Assimilation, dissimilation, and detoxification of formaldehyde, a central metabolic intermediate of methylotrophic metabolism. Chem Record 5(6):367–375. 10.1002/TCR.20056 16278835 10.1002/tcr.20056

[CR148] Yurimoto H, Komeda T, Lim CR et al (2000) Regulation and evaluation of five methanol-inducible promoters in the methylotrophic yeast *Candida boidinii*. Biochimica et Biophysica Acta (BBA) - Gene Structure and Expression 1493(1–2):56–63. 10.1016/S0167-4781(00)00157-3 10978507 10.1016/s0167-4781(00)00157-3

[CR149] Yurimoto H (2009) Molecular basis of methanol-inducible gene expression and its application in the methylotrophic yeast *Candida boidinii*. Biosci Biotechnol Biochem 73(4):793–800. 10.1271/BBB.80825 19352035 10.1271/bbb.80825

[CR150] Zavec D, Troyer C, Maresch D et al (2021) Beyond alcohol oxidase: The methylotrophic yeast *Komagataella phaffii* utilizes methanol also with its native alcohol dehydrogenase Adh2. FEMS Yeast Res 21(2):foab009. 10.1093/femsyr/foab009 33599728 10.1093/femsyr/foab009 PMC7972947

[CR151] Zhai X, Ji L, Gao J et al (2021) Characterizing methanol metabolism-related promoters for metabolic engineering of *Ogataea polymorpha*. Appl Microbiol Biotechnol 105(23):8761–8769. 10.1007/s00253-021-11665-5 34748038 10.1007/s00253-021-11665-5

[CR152] Zhai X, Gao J, Li Y et al (2023) Peroxisomal metabolic coupling improves fatty alcohol production from sole methanol in yeast. Proc Natl Acad Sci U S A 120(12):e2220816120. 10.1073/pnas.2220816120 36913588 10.1073/pnas.2220816120 PMC10041095

[CR153] Zhang W, Bevins MA, Plantz BA, et al (2000) Modeling *Pichia pastoris* growth on methanol and optimizing the production of a recombinant protein, the heavy-chain fragment C of botulinum neurotoxin, serotype A. Biotechnol Bioeng 70(1):1–8. 10.1002/1097-0290(20001005)70:1%3C;1::AID-BIT1%3E;3.0.CO;2-Y 10.1002/1097-0290(20001005)70:1<1::aid-bit1>3.0.co;2-y 10940857

[CR154] Ziegler AL, Ullmann L, Boßmann M et al (2024) Itaconic acid production by co-feeding of *Ustilago maydis*: a combined approach of experimental data, design of experiments, and metabolic modeling. Biotechnol Bioeng 121(6):1846–1858. 10.1002/bit.28693 38494797 10.1002/bit.28693

