# Correction: Yeast Midas’ touch: recent advances in the valorisation of methanol by yeasts

**DOI:** 10.1007/s11274-025-04685-0

**Published:** 2025-11-18

**Authors:** Alessandra Mauri, Lorenzo García Tejada, Lars M. Blank

**Affiliations:** 1 Institute of Applied Microbiology (iAMB), RWTH Aachen University, Aachen, Germany; 2 Corbion Innovation Center, Gorinchem, Netherlands; 3 WSS Research Centre “Catalaix”, Aachen, Germany

**Correction: World Journal of Microbiology and Biotechnology**

10.1007/s11274-025-04642-x

In the original publication of the article the sentence “Endowed with peculiar physiology, these can utilise the reduced C1 molecule as their sole caglycerol can derepress the methanol oxidase promoterrbon and energy source.” has been published incorrectly in the abstract section. The correct sentence should read as:

“Endowed with peculiar physiology, these can utilise the reduced C1 molecule as their sole carbon and energy source.”

The original article has been corrected.

